# Genome-Scale Genetic Interactions and Cell Imaging Confirm Cytokinesis as Deleterious to Transient Topoisomerase II Deficiency in *Saccharomyces cerevisiae*

**DOI:** 10.1534/g3.117.300104

**Published:** 2017-08-23

**Authors:** Cristina Ramos-Pérez, Jessel Ayra-Plasencia, Emiliano Matos-Perdomo, Michael Lisby, Grant W. Brown, Félix Machín

**Affiliations:** * Unidad de Investigación, Hospital Universitario Nuestra Señora de Candelaria, 38010 Santa Cruz de Tenerife, Spain; † Universidad de la Laguna, 38200 San Cristóbal de La Laguna, Santa Cruz de Tenerife, Spain; ‡ Department of Biology, University of Copenhagen, DK-2200, Denmark; § Department of Biochemistry and Donnelly Centre, University of Toronto, Ontario M5S3E1, Canada

**Keywords:** topoisomerase II, *top2-4* and *top2-5*, synthetic genetic array analysis, mitotic exit network, cytokinesis, plasma membrane abscission, anaphase bridges, Cdc14

## Abstract

Topoisomerase II (Top2) is an essential protein that resolves DNA catenations. When Top2 is inactivated, mitotic catastrophe results from massive entanglement of chromosomes. Top2 is also the target of many first-line anticancer drugs, the so-called Top2 poisons. Often, tumors become resistant to these drugs by acquiring hypomorphic mutations in the genes encoding Top2. Here, we have compared the cell cycle and nuclear segregation of two coisogenic *Saccharomyces cerevisiae* strains carrying *top2* thermosensitive alleles that differ in their resistance to Top2 poisons: the broadly-used poison-sensitive *top2-4* and the poison-resistant *top2-5*. Furthermore, we have performed genome-scale synthetic genetic array (SGA) analyses for both alleles under permissive conditions, chronic sublethal Top2 downregulation, and acute, yet transient, Top2 inactivation. We find that slowing down mitotic progression, especially at the time of execution of the mitotic exit network (MEN), protects against Top2 deficiency. In all conditions, genetic protection was stronger in *top2-5*; this correlated with cell biology experiments in this mutant, whereby we observed destabilization of both chromatin and ultrafine anaphase bridges by execution of MEN and cytokinesis. Interestingly, whereas transient inactivation of the critical MEN driver Cdc15 partly suppressed *top2-5* lethality, this was not the case when earlier steps within anaphase were disrupted; *i.e.*, *top2-5 cdc14-1*. We discuss the basis of this difference and suggest that accelerated progression through mitosis may be a therapeutic strategy to hypersensitize cancer cells carrying hypomorphic mutations in *TOP2*.

Upon chromosome replication, topological intertwining arises between sister chromatids. These intertwines often become interlocked (*i.e.*, catenations) due to the confinement of the very long chromosomes in the reduced space of the nucleus. Catenations preclude sister chromatid segregation in anaphase, and the key enzyme in all life forms for removing them is topoisomerase II (Top2) (Nitiss 2009a; Vos *et al.* 2011). Top2 works by making transient double-strand breaks (DSBs) on one chromatid, allowing the passage of its sister through this break. Importantly, a human homolog of Top2, hTOPOIIα, is the main target of first-line anticancer drugs including etoposide and doxorubicin (Deweese and Osheroff 2009; Nitiss 2009b). These drugs trap Top2-mediated DSBs and are called Top2 poisons. The resulting DSBs are more abundant and less efficiently repaired in cancer cells than in normal cells and this, in turn, leads to the selective killing of the tumor. Human TOPOIIα is often mutated and/or downregulated during acquisition of secondary resistance to Top2 poisons, and this fact could be exploited for second-line anticancer treatments (Larsen *et al.* 2003; Nitiss 2009b; Holohan *et al.* 2013).

Top2 is essential for cellular viability. In unicellular eukaryotes and bacteria the study of Top2 functions has been largely facilitated by the availability of conditional alleles. In the yeasts *Saccharomyces cerevisiae* and *Schizosaccharomyces pombe* early studies showed that inactivation of Top2 by means of thermosensitive (ts) alleles leads to a mitotic catastrophe as determined by a sudden loss of viability once the cells reach anaphase (Holm *et al.* 1985; Uemura and Tanagida 1986). In agreement with a role in removing sister chromatid catenations, Top2 inactivation yielded cells with DAPI-stained anaphase bridges and broken chromosomes once cells completed cytokinesis (DiNardo *et al.* 1984; Uemura and Yanagida 1984; Holm *et al.* 1985, 1989; Uemura and Tanagida 1986). In the case of *S. cerevisiae*, all these studies were carried out with two ts alleles isolated in independent screens, *top2-1* and *top2-4*. Both alleles yield Top2-ts proteins sensitive to poisons. In the same screen where *top2-4* was isolated, *top2-5* was also obtained (Holm *et al.* 1985). Later, *top2-5* was shown to be resistant to poisons and served as a key tool to understand the mechanism of action of this class of clinical drugs (Jannatipour *et al.* 1993; Perego *et al.* 2000). Nevertheless, the cell cycle of the *top2-5* strain was not characterized and has been assumed to be equivalent to that of the *top2-4* strain.

Here, we have revisited the cell cycle progression of cells expressing the broadly used *top2-4* allele and compared its behavior to a coisogenic *top2-5* strain. In addition, we have performed a genome-scale synthetic genetic array (SGA) analysis for these two *top2-ts* alleles. We show that *top2-5* goes faster through the cell cycle and gathers more genetic interactions related to mitotic progression than *top2-4*. In addition, we show that execution of the mitotic exit network (MEN) has specific deleterious effects on sublethal downregulation of Top2-5, and that this correlates with destabilization of anaphase bridges by cytokinesis.

## Materials and Methods

### Yeast strain construction, cell cycle experiments, and fluorescence microscopy

All the strains used in this work are listed in Supplemental Material, Table S1 in File S1 together with their relevant genotypes. C-terminal tagging with GFP/RFP variants or an auxin-based degron system, gene deletions and ts allele transfers were carried out using standard PCR methods as described before (Tong *et al.* 2001; Janke *et al.* 2004; Nishimura *et al.* 2009).

Most strains were grown overnight in air orbital incubators at 25° in YPD media before every experiment. Cell cycle time course experiments and fluorescence microscopy were performed as described before (Quevedo *et al.* 2012; Silva *et al.* 2012; García-Luis and Machín 2014). Briefly, asynchronous cultures of *MAT***a** haploids were adjusted to OD_600_ = 0.3 and then synchronized in G1 at 25° for 3 hr by adding 50 ng/ml (*bar1*Δ strains) or 5 μg/ml (*BAR1* strains) of α-factor (T6901, Sigma-Aldrich). The G1 release was induced by washing the cells twice in YPD and resuspending them in fresh media containing 0.1 mg/ml of pronase E (81750, Sigma-Aldrich). Next, the culture was incubated at 37° for 4 hr and samples were taken every 30 min for direct visualization under a Leica DMI6000 fluorescence microscope. DNA was stained using DAPI (32670, Sigma-Aldrich) at 4 μg/ml final concentration after keeping the cell pellet 24 hr at −20°. In the experiments performed to visualize ultrafine anaphase bridges (UFBs), a synthetic complete medium containing 100 µg/ml adenine (SC+Ade) was used instead of YPD and images were taken with a Zeiss AxioImager Z1 fluorescence microscope.

Plasma membrane (PM) abscission and zymolyase digestion were employed to address progression of cytokinesis (Norden *et al.* 2006; Mendoza *et al.* 2009; Quevedo *et al.* 2012). For membrane abscission, the PM reporter 2·PH-GFP (two GFP-fused pleckstrin homology domains of phospholipase C from *Rattus norvegicus*) was used. When this reporter was not available, PM was stained with 5 µg/ml Hoechst 33258 (94403, Sigma-Aldrich) for either 5 or 15 min at 37° before processing for fluorescence microscopy. Aside from the DNA, Hoechst dyes have great affinity for the lipid bilayer where they also become strongly fluorescent (Shapiro and Ling 1995). Zymolyase treatment was performed as described before (Quevedo *et al.* 2012). Briefly, samples were taken from the culture, fixed with 5% formaldehyde for 1 hr at 37°, and then washed twice with PBS and once with 1 M sorbitol in 50 mM KPO_4_, pH 7.5. Finally, the sample was split in two; one half was treated with 0.2 mg/ml zymolyase 20T (E1005, Zymo Research) in the above sorbitol buffer containing 4 mM β-mercaptoethanol for 20 min at 37°, whereas the other half was treated in the same conditions but without zymolyase (mock control).

For the time-lapse movies, an asynchronous culture was concentrated by centrifugation to three OD_600_ equivalents and plated on YPDA (YPD, agar 2% w/v). Patches were made from this plate and mounted on a microscope slide. They were incubated at 37° in high humidity chambers to avoid drying of the agarose patch. Photos were taken every 30 or 60 min for 6 hr in order to minimize both photobleaching and cell damage by the excitation light.

For clonogenic assays,∼300 cells (as calculated from OD_600_ measurements) from an asynchronous culture were seeded on two YPD plates. One plate was incubated at 25° for 3 d, whereas the other was first preincubated at 37° for 6 hr before being transferred to 25°. Proportion of survivors was calculated from the fraction of colonies that grew after the 37° preincubation normalized to the number of colonies on plates continuously grown at 25°. The same principle of temperature incubations was employed in the spot dilution assay. In this case, the asynchronous culture was first normalized to OD_600_ = 1, then 1:10 serial diluted, and finally spotted on YPD plates (∼5 μl per spot) with a 48-pin replica plater (R2383, Sigma-Aldrich).

Variation between independent experiments was evaluated by SEM. Comparisons between mean values for different strains or conditions were performed by the unpaired two-sided student’s *t*-test. When percentages were calculated for selected cell phenotypes observed by microscopy, exact binomial 95% confidence intervals (CI95) were also included as error bars in order to inform about the accuracy of such observation. When percentages are mentioned in the main text, CI95 is indicated afterward in parentheses. Cells from at least two independent experiments were pooled for such calculations.

### Synthetic genetic array analyses

SGA was performed as described before (Tong *et al.* 2001; Baryshnikova *et al.* 2010; Li *et al.* 2011). In order to make the strain arrays (described in detail in Figure S1 in File S1), we first replaced the *TOP2* locus in the haploid *MATα* strain Y7092 with our query *top2-ts* alleles attached to the selection marker *natMX4* (resistance to nourseothricin). For consistency, we also attached the *natMX4* marker to our reference *TOP2* Y7092 strain. The new *natMX4* strains were then mated with the *MAT***a** mutant collections (4322 knockout strains for nonessential genes plus 1231 strains with thermosensitive alleles for essential genes). These panels of *MAT***a** strains bear the *kanMX4* marker (resistance to G418) at the mutated locus. Diploids were selected on YPD plates containing both nourseothricin and G418, and later sporulated and selected for *MAT***a** haploids containing both markers. Once the *TOP2*, *top2-4*, and *top2-5* arrays were constructed they were replicated on to plates with the same medium used in the *MAT***a** selection and exposed to the different temperature regimes described in the *Results* section.

The image analysis, processing, and fitness scoring of the arrays were done using SGAtools (http://sgatools.ccbr.utoronto.ca/about) (Wagih *et al.* 2013). Gene Ontology (GO) enrichment analysis was performed using the Generic GO Term Finder (http://go.princeton.edu/cgi-bin/GOTermFinder) of Princeton University (Boyle *et al.* 2004). Networks were made with Cytoscape v3.3.0 (http://www.cytoscape.org/cy3.html) (Shannon *et al.* 2003).

### Data availability

Strains are available upon request. File S1 contains additional material and methods, four supplemental figures, six supplemental tables, and legends for the SGA files included in File S2. File S2 is a zip compressed file which contains 12 Microsoft Excel files with raw and processed data for each SGA analysis.

## Results

### Three nuclear segregation patterns can be revealed by live microscopy of top2-4 and top2-5 cells

We started this work by revisiting fluorescence microscopy time course experiments in the original *top2-4* and *top2-5* ts strains (Holm *et al.* 1985). As a control, we also included the *TOP2* reference wild-type allele in the same genetic background. We first engineered the strains in order to label the histone H2A (*HTA2* gene) with GFP. This strategy allowed us to complement the time course experiments with fluorescence videomicroscopy of the nuclear DNA without adding DNA intercalating dyes. We further labeled Rad52 with RedStar2 to assess DNA damage upon inactivation of Top2. Rad52 forms nuclear foci to repair DSBs through the homologous recombination repair pathway (Lisby *et al.* 2001).

**Figure 1:**
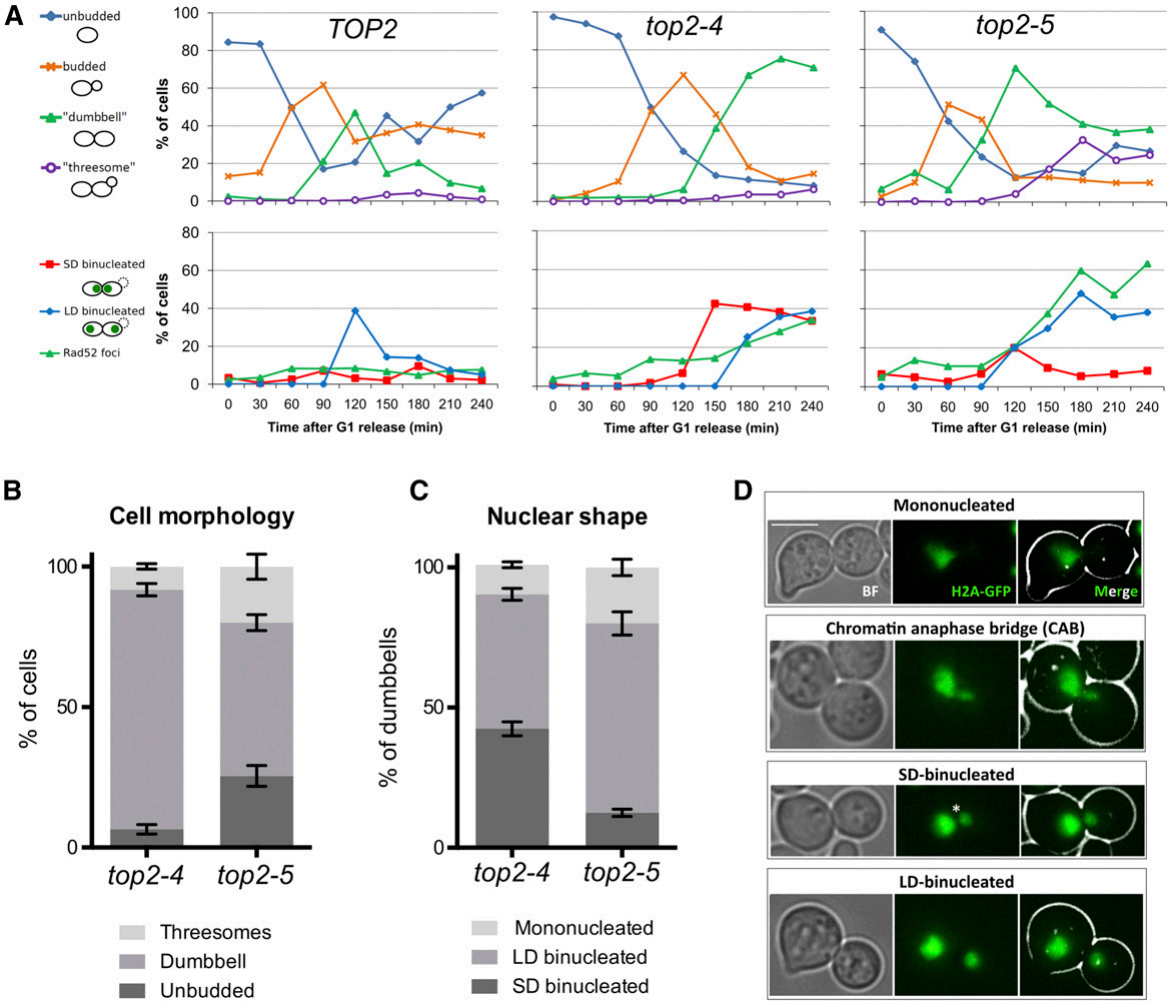
Cell morphology and segregation of the nucleus upon inactivation of Top2 by the temperature-sensitive *top2-4* and *top2-5* alleles. (A) *HTA2-GFP Rad52-RedStar2* yeast cells carrying different alleles of the *TOP2* gene (wild-type *TOP2* and thermosensitive alleles *top2-4* and *top2-5*) were synchronized in G1 at the permissive temperature (25°) and then released into a synchronous cell cycle at 37° for 4 hr. Samples were taken every 30 min and directly analyzed by fluorescence microscopy. More than 150 cells were counted for each time point. Upper charts depict the budding pattern of the population. Lower charts depict the percentage of cells with histone-labeled split nuclear masses that either remained at a short distance (≤1 µm) from one another (“SD-binucleated”) or were separated by a longer distance (“LD-binucleated”). Percentage of cells with at least one Rad52 focus is also included. (B) Stacked bar chart of the observed end-point budding morphologies from three independent experiments (after 4 hr at 37°, mean ± SEM). (C) Stacked bar chart of the nuclear morphologies observed for the dumbbell subpopulation from (B). (D) Examples of different nuclear morphologies observed in cell dumbbells of *top2-ts* mutants. The asterisk highlights that SD-binucleated cells have an apparent segregation defect, yet a proper chromatin anaphase bridge (CAB) cannot be visualized. The bar corresponds to 5 μm.

All strains were arrested in G1 at the permissive temperature (25°) for 3 hr and then released at 37° to follow the progression through a synchronous cell cycle. As expected, the *TOP2* control cycled normally after the release, with segregation of the nuclear masses taking place very quickly at 90–120 min and no signs of DNA damage after that (Figure 1A, *left panels*). Separation between the segregated histone-labeled masses was clear and often laid close to the cell poles. Hereafter, we refer to this segregation phenotype as long-distance binucleated (LD-binucleated) and grouped cells within this phenotype provided that the distance between the split masses was >1 μm. From 150 min onwards, the *TOP2* strain split the daughter from the mother and asynchronously entered a second cell cycle. In contrast to *TOP2*, the *top2-4* mutant exhibited a phenotype of cells stuck in anaphase after 4 hr, in which ∼75% of the cells were in a “dumbbell” state (*i.e.*, the bud as big as the mother) and ∼35% (50% of dumbbells) had two very close nuclear masses as determined by histone labeling (Figure 1A, *central panels*; Figure 1, B and C). We refer to this abnormal form of nuclear segregation as short-distance binucleated (SD-binucleated, <1 μm of separation) (Figure 1D). Unexpectedly, the actual presence of chromatin anaphase bridges (CABs, *i.e.*, stretched histone-labeled DNA across the bud neck, Figure 1D) was low, with a SD-binucleated:CAB ratio of 5:1. The presence of SD-binucleated was constant from 150 min onwards. Coinciding with this change in the nuclear morphology, a steady increase of cells with Rad52 foci was also observed (up to 35% of cells by 240 min). In the case of *top2-5*, we observed a quicker G1-S entry and a different mix of cell morphologies by the end of the time course (Figure 1A, *right panels*; Figure 1B). Thus, we found a decrease of the dumbbell category from 150 min and the presence of “threesomes,” where the mother cell has rebudded, in up to 25% of the population, a percentage that was higher than that of *top2-4* (Figure 1B). Furthermore, there was only a transient peak of SD-binucleated at 120 min (Figure 1A, *right panels*), with <10% of the cells having this morphology after 4 hr (Figure 1B). Likewise, Rad52 foci abruptly rose from 120 min, reaching 60% of all cells by 180 min, twice as many as in *top2-4* (Figure 1A, *lower panels*).

**Figure 2:**
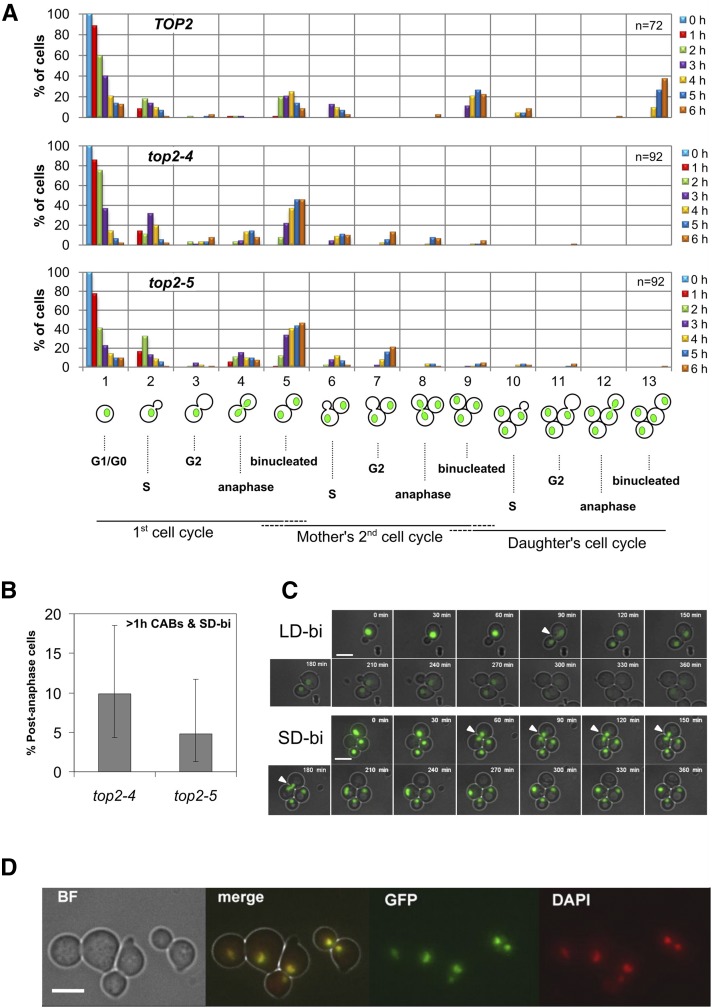
Single-cell live microscopy of *top2-4* and *top2-5* alleles for the first two cell cycles at the restrictive temperature. (A) *HTA2-GFP* cells carrying different alleles of the *TOP2* gene (*TOP2*, *top2-4* and *top2-5*) were grown at 25°, concentrated to OD_600_ = 3, spread onto YPD agarose patches, and filmed under the microscope at 37° for 6 hr, taking images every 1 hr. Several films were independently recorded and the subpopulation of cells which were at G1 (unbudded) at the time of the temperature shift were followed (cell number is indicated on the upper right corner of each bar chart). Each hour, cells were either kept in their preceding category (starting at “1,” first cell cycle G1) or moved into one of the 13 categories shown on the *X*-axis. The bar charts represent the percentage of cells in each of these categories at any given time point. Horizontal dashed line ends under the categories indicate uncertainty about which cell cycle stage cells are in (*e.g.*, category “5” grouped those cells which are in the first cell cycle’s telophase together with those mother and daughter cells in the second cycle’s G1). (B) Analysis of cells where anaphase lasted longer than 1 hr in the cells studied in (A); *i.e.*, cells that remained in category “4” for at least two successive frames. Only cells that reached or passed the first anaphase were considered for the analysis. Error bars represent CI95 of the cell proportion. (C) The same *top2-ts HTA2-GFP* strains used for (A) were filmed again at 37° but taking images every 30 min. Representative cells where a CAB was seen are shown. The upper “LD-binucleated” example is from a *top2-5* cell and the lower “SD-binucleated” example is taken from a *top2-4* cell. White-filled triangles pointing to H2A-GFP CABs are included in the corresponding frames. (D) *HTA2-GFP top2-4* after 4 hr at 37° in liquid cultures showing a perfect colocalization of H2A-GFP and DAPI staining in cells with the SD-binucleated morphology. The bar corresponds to 5 μm. BF, bright field.

We next complemented the time course experiments with videomicroscopy aimed to follow up single cells throughout their first and second cell cycles (up to 6 hr). In order to do so, we filmed on agarose patches an asynchronous population of cells at the restrictive temperature and analyzed those cells that were in G1 (unbudded) at the time of the temperature shift. First, we filmed the *TOP2* strain as a control and found normal cell cycle progression for up to two generations for the mother and one generation for its first daughter (Figure 2A, upper chart). We also found little indication of cells undergoing arrest in G1 (categories 1, 5 and 9), in G2/M (categories 3, 7 and 11), or presenting CABs (categories 4, 8 and 12). By contrast, we found that *top2-ts* cells starting in G1 struggled to rebud in the second cell cycle (Figure 2A, mid and lower charts), with only a minority of cells that had done so by 6 hr (∼20% of *top2-4* and *top2-5* mothers; *i.e.*, the sum of the orange bar values from morphological categories 6–13). As in the case of the time course with liquid cultures, the cell cycle on agarose patches was slightly quicker in *top2-5* than in *top2-4* (drop of category 1).

Videomicroscopy allowed us to gain insights about the time that cells spent with the SD-binucleated and CABs phenotypes (Figure 2B). Thus, around 5–10% of *top2-ts* cells had these phenotypes in two continuous frames (*i.e.*, aberrant segregation phenotypes lasted >1 hr). Although the low percentage and limited number of single cells analyzed precluded a definitive assessment, these long-lasting incomplete segregations occurred in *top2-4* cells more often. A close look at cells while transiting through anaphase (30 min frame intervals) confirmed the long-lasting nature of these “short distance” nuclear morphologies, as opposed to those cells that quickly ended up in the LD-binucleated morphology (Figure 2C). To confirm that the SD-binucleated phenotype was a proper split of nuclear masses rather than a mere relocalization of histones, we took samples and stained the DNA with DAPI. In all cases, the DAPI signal overlapped with the H2A-GFP (Figure 2D).

Finally, the use of live cell imaging also allowed us to visualize other striking aberrant anaphases. Although these phenotypes occurred in <1% of filmed cells, both G1 and early S phase (small bud) at the time of the temperature shift, they show remarkable instances of uncoupling between the nuclear division and the cell cycle in *top2-ts* (*e.g.*, rebudding before splitting the chromatin bridge) (Figure S2 in File S1).

### Cytokinesis progression correlates with the shape of the segregating nuclear mass in top2-ts mutants

Both the presence of dynamic SD-binucleated phenotypes and the low percentage of CABs were surprising for *top2-ts*, taking into account that depletion of Top2 is considered the prototypical model to elicit anaphase bridges. This led us to study *top2-ts* anaphases in more detail. First, we wondered about the completion of cytokinesis in these mutants. Previous reports have suggested that *top2-4* CABs delay cytokinesis (Mendoza *et al.* 2009). This delay would explain the observed accumulation of dumbbells and threesomes, but it would also predict an easy visualization of CABs due to the gross defects in sister chromatid resolution.

**Figure 3:**
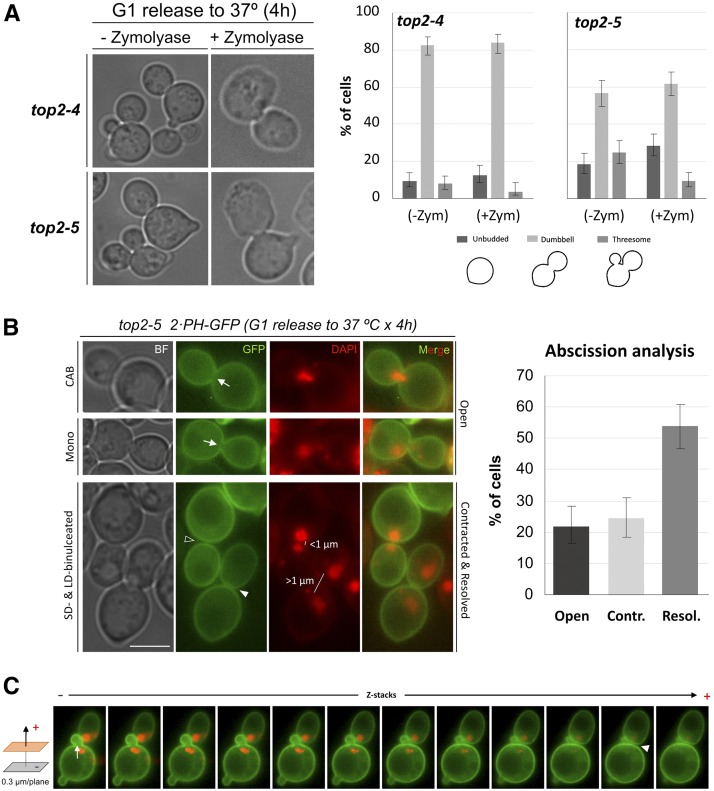
Cytokinesis defects in *top2-ts* and correlation with the different abnormal nuclear segregation morphologies. (A) The same *top2-4* and *top2-5* strains used for Figure 2 were synchronized in G1 at the permissive temperature (25°) and then released into a synchronous cell cycle at 37° for 4 hr. A sample was then taken, fixed with formaldehyde, and split in two for addressing cytokinesis through the cell wall digestion assay; *i.e.*, comparing cell morphologies after zymolyase or a mock treatment. On the left, representative pictures of cells for the mock control and zymolyase treatment. On the right, quantification of major cell morphologies. Error bars depict CI95 of the measured percentages. (B) A *top2-5* strain expressing the plasma membrane (PM) marker PH-GFP was release in a synchronous cell cycle as in (A). A sample taken after 4 hr was stained with DAPI and visualized under the microscope. On the left, representative cells showing different degrees of cytokinetic completion and their best corresponding nuclear shape across the neck. The arrow points to an “open” neck (no PM ingression) with either a CAB across (first cell) or mononucleated (second cell), the open arrowhead points to a “resolved” membrane at the neck (*i.e.*, abscission with septum deposition in between) with a SD-binucleated nuclear morphology, and the filled arrowhead points to a “contracted” PM. (C) A complete Z-stack series of a representative threesome is shown. Note how the mother and the daughter have a resolved PM at the neck (filled arrowhead), whereas the mother and its second bud have an open neck (white arrow). The bar corresponds to 5 μm. BF, bright field.

Cell wall digestion has often been used to check whether mother and daughter cells have finished cytokinesis yet not completed septum formation. We performed this digestion on *top2-ts* cells that were well into anaphase or beyond; *i.e.*, 4 hr after the G1 release at 37° (Figure 3A). Strikingly, dumbbells remained as such, although more than half of the threesomes were split in two (one budded and one unbudded cell, whose proportions thus became slightly higher upon digestion). This result was somewhat expected for *top2-4*, based on the higher prevalence of SD-binucleated dumbbells and previous reports (Mendoza *et al.* 2009); however, it was surprising for *top2-5* since dumbbells were mostly LD-binucleated and were steadily being split into two by 4 hr (Figure 1, A and B). Hence, we decided to complete the analysis of *top2-5* by directly checking abscission of the PM at the bud neck. In order to do so, we engineered the original *top2-5* strain to express the PM marker 2xPH-GFP. Interestingly, abscission (*i.e.*, PM resolved in two at the bud neck) had taken place in at least 50% of the dumbbells and all threesomes 4 hr after the G1 release (Figure 3, B and C). As for the other 50% of dumbbells that fell into a preabscission category, half of them had a full contracted furrow and the other half an open neck; *i.e.*, PM ingression at the cleavage furrow was absent or just partial. Interestingly, CABs were only visible in cells with an open cleavage furrow, although most of these cells were just mononucleated (∼1:2 ratio). By contrast, SD-binucleated cells always had either a contracted or a resolved PM (Figure 3B, photos).

### Chromatin anaphase bridges in top2-ts are stabilized by preventing mitotic exit

Since there is PM ingression in *top2-ts* mutants, we next decided to check what the nuclear morphology looks like if ingression is fully abrogated. Cytokinesis is executed by the MEN, which also makes possible the telophase-to-G1 transition (Jaspersen *et al.* 1998; Meitinger *et al.* 2012; Weiss 2012). We chose two broadly-used ts alleles for essential genes involved in MEN to prevent cytokinesis, *cdc15-2* and *cdc14-1*. At 37°, both mutants block cells in telophase with an open cleavage furrow (Bembenek *et al.* 2005); however, there are important differences between them. Cdc14 is the key MEN player, but it also has physiological roles unrelated to cytokinesis in early anaphase. Thus, Cdc14 gets activated twice in anaphase, first by the so-called FEAR network and then by MEN (Stegmeier and Amon 2004; Machín *et al.* 2016). Cdc15 is only involved in the Cdc14 activation by MEN. Importantly, whereas the single *cdc15-2* mutant allows full segregation of sister chromatids, single *cdc14-ts* mutants give rise to a thin CAB that comprises the ribosomal DNA (rDNA) chromosome arm (D’Amours *et al.* 2004; Machín *et al.* 2005).

**Figure 4:**
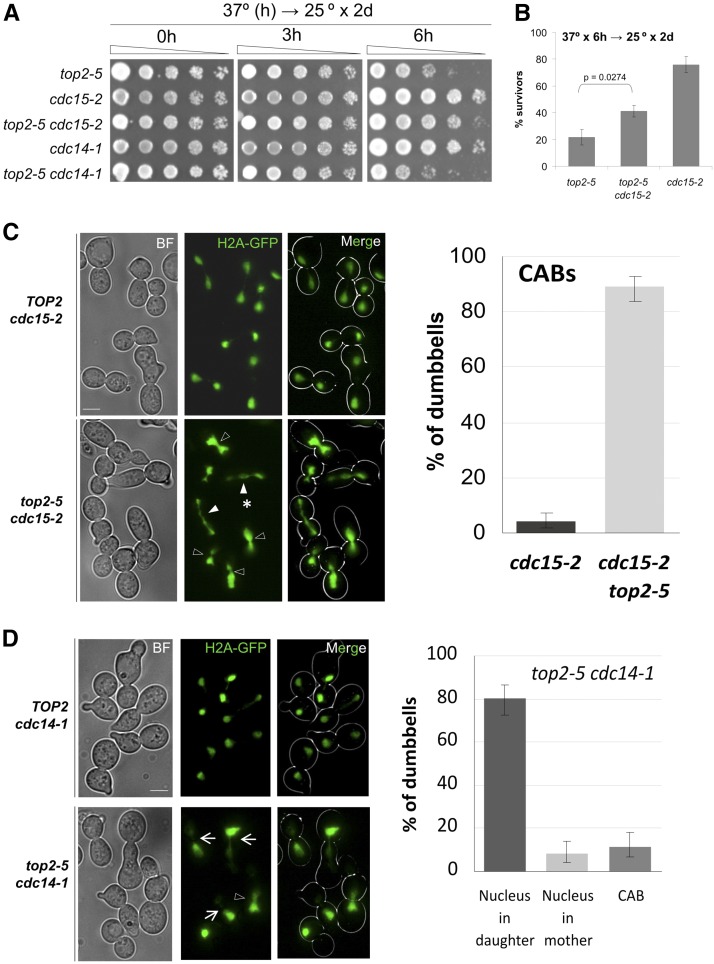
The mitotic exit network (MEN) destabilizes *top2*-mediated chromatin anaphase bridges (CABs). (A) Spot dilution assay to compare survivability of different combinations of the *top2-5*, *cdc14-1*, and *cdc15-2* alleles to a transient temperature shift to 37°. All strains are *HTA2-GFP bar1*∆ derivatives from the corresponding *TOP2* and *top2-5* coisogenic strains. (B) Clonogenic assay to compare survivability of the *top2-5*, *cdc15-2*, and *top2-5 cdc15-2* strains to a transient 6 hr shift to 37° (mean ± SEM, *n* = 5). (C) The *cdc15-2 HTA2-GFP* and *top2-5 cdc15-2 HTA2-GFP* strains were synchronized in G1 at the permissive temperature (25°) and then released into a synchronous cell cycle at 37° for 4 hr. Samples were taken at the end of the experiment and analyzed by fluorescence microscopy. On the left, representative microscope fields for each strain. On the right, bar chart of cells with CABs (error bars are CI95). Open arrowheads point to examples of short CABs (∼1/3 of observed CABs in *top2-5 cdc15-2*). Filled arrowheads point to examples of long CABs (∼2/3 of observed CABs). The asterisk highlights the complex strung chromatin bridges seen in ∼50% of the long CABs. (D) The *cdc14-1 HTA2-GFP* and *top2-5 cdc14-1 HTA2-GFP* strains were treated as in (C). On the left, representative microscope fields for each strain. On the right, bar chart of nuclear morphologies in dumbbells (error bars are CI95). Arrows point to cells where the bulk of the nucleus migrated to the bud, whereas open arrowheads point to CABs. Arrows and arrowheads point exactly at the bud neck. The bar corresponds to 5 μm. BF, bright field.

First, we constructed *top2-5 cdc15-2* and *top2-5 cdc14-1* double mutants and checked whether they were better protected than the single *top2-5* mutant against transient Top2 inactivation. Protection by *cdc15-2* has been reported before for Top2 depletion through a degron allele (Baxter and Diffley 2008). We found that *top2-5 cdc15-2* survived slightly better than *top2-5* in the restrictive regime (Figure 4, A and B), whereas there was no difference for *top2-5 cdc14-1*. In order to address the nuclear morphology, we arrested these strains in telophase, together with the corresponding *TOP2cdc15-2/cdc14-1* controls. We found that *top2-5 cdc15-2* could not resolve the CAB (Figure 4C). By contrast, full segregation of the histone signal was seen in all *TOP2cdc15-2* cells. In the case of *top2-5 cdc14-1*, the nuclear segregation was worse; we observed that most of the nucleus was in the bud (Figure 4D). This synergistic nuclear segregation defect in *top2-5 cdc14-1* may account for the lack of genetic suppression of *top2-5* despite *cdc14-1* also blocking cytokinesis (see *Discussion* chapter). Overall, these two double mutants demonstrate that the *top2-5* strain gives rise to actual CABs and that the contraction of the cytokinetic furrow quickly split this CAB apart (see below).

We also made a *top2-4 cdc15-aid* strain. We used *cdc15-aid* (Cdc15 depletion by auxin addition rather than temperature shift) because it was difficult to phenotypically confirm *top2-4 cdc15-2* as *top2-4* alone got stuck as dumbbells (Figure 1). We also observed CABs when both Top2-4 and Cdc15-aid were depleted [33% (24–43%)]. In this case, most CABs had a SD-binucleated appearance (Figure S3 in File S1).

### Ultrafine anaphase bridges are also split apart in top2-5

**Figure 5:**
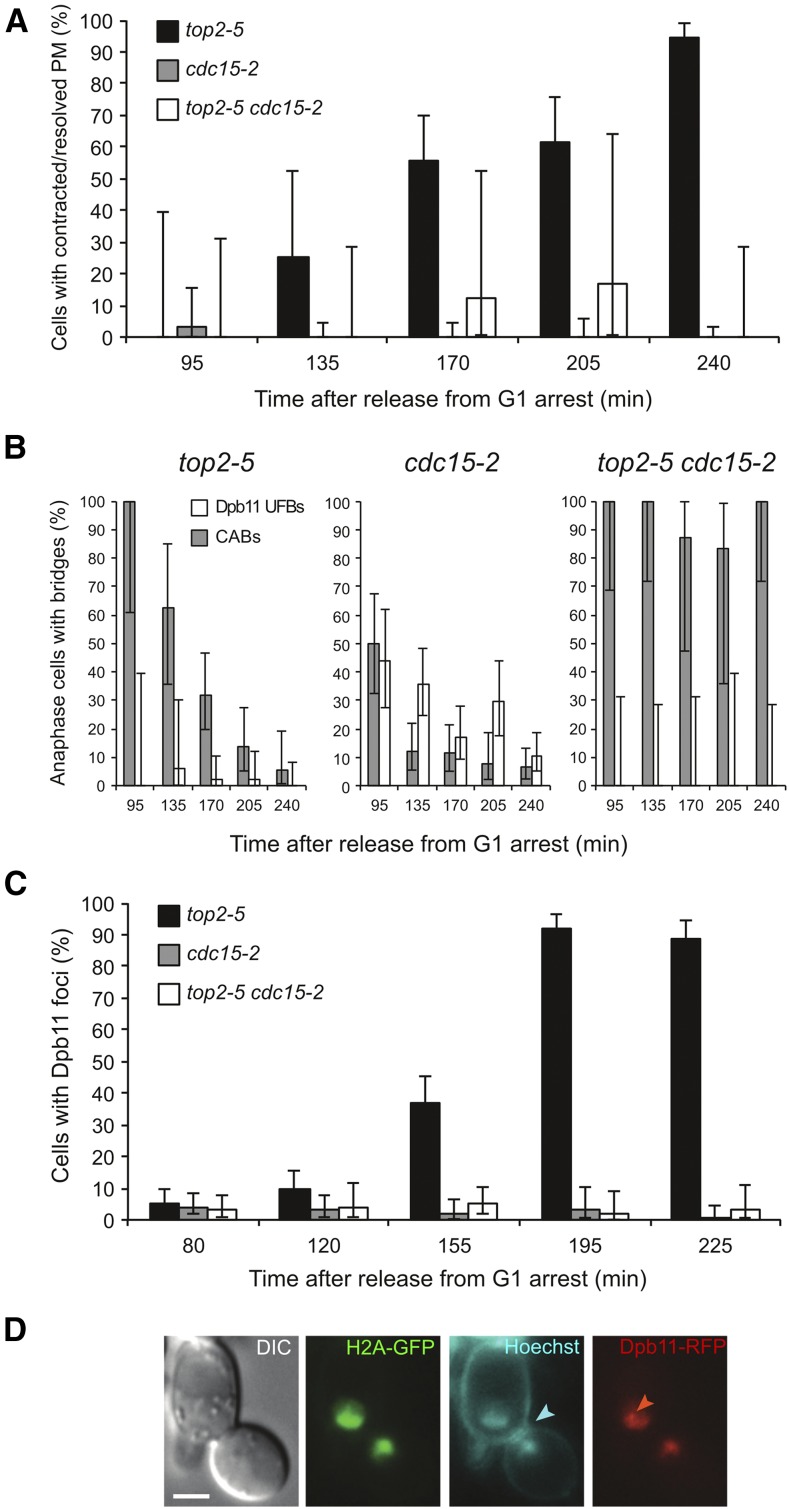
Dpb11 Ultrafine bridges are also destabilized by cytokinesis in *top2-5*. Coisogenic *top2-5*, *cdc15-2*, and *top2-5 cdc15-2* strains bearing the *HTA2-GFP Dpb11-yEmRFP bar1*∆ genotype were released into a synchronous cell cycle at 37°. At the indicated time points, the plasma membrane (PM) was stained with 5 µg/ml Hoechst 33258 for 5 min at 37° before processing for fluorescence microscopy. (A) The time course of PM abscission (full contraction and resolution at the cytokinetic plane). Note how *top2-5* mutant cells progress to abscission. (B) The time course of both CABs and UFBs in anaphase cells. Note how *top2-5* mutant cells do not accumulate UFBs, not even transiently, while CABs are split apart. (C) The time course of Dpb11 foci formation. Note how *top2-5* mutant cells accumulate Dpb11 foci. Error bars depict CI95 (*n* = 50–200). (D) The prototypical example of a late anaphase *top2-5* cell; *i.e.*, PM has completed abscission, there is no longer a CAB connecting the mother and the daughter cell, nor are there any Dpb11-UFBs, and Dpb11 accumulates in foci within the split nuclear masses instead. The cyan arrowhead points to the resolved PM, whereas the red arrowhead points to a Dpb11 focus. The bar corresponds to 3 μm. DIC, differential interference contrast.

A final cell imaging analysis we performed was to check UFBs in *top2-5* and address their fate after membrane abscission. These UFBs comprise mysterious forms of DNA that connect segregated nuclei and are refractory to classical DNA dyes and histone labeling (Chan *et al.* 2007). UFBs can, however, be detected with specific proteins that interact with them in anaphase; one such protein in yeast is Dpb11 (Germann *et al.* 2014). Thus, we looked at a *top2-5* strain that had been triple-labeled with H2A-GFP, Dpb11-RFP, and the PM reporter Hoechst 33258, which becomes fluorescent in the CFP channel when bound to membranes (Shapiro and Ling 1995); we confirmed that Hoechst 33258 can also stain yeast PM *in vivo* and that a very short incubation (5 min) minimizes costaining of the DNA (Figure S4 in File S1). In addition, we included a *top2-5 cdc15-2* strain to prevent cytokinesis, as well as the corresponding *TOP2cdc15-2* control. First, we again observed PM abscission in *top2-5* at late time points of a synchronous cell cycle at 37°, whereas PM abscission was mostly absent in the *cdc15-2* strains (Figure 5A). Second, we again observed how CABs dropped as *top2-5* cells complete abscission, whereas CABs remained throughout the time course in the *top2-5 cdc15-2* strain (Figure 5B). Interestingly, few Dpb11-stained UFBs could be detected in either strain, whereas UFBs were relatively common in the *TOP2cdc15-2* strain (30–40% of cells). Since there was no transient enrichment of UFBs relative to CABs during the drop of the latter in the *top2-5* strain, we conclude that UFBs are likely to be severed by PM abscission in this mutant. Moreover, we found a relocalization of Dpb11 to foci, which was specific for the *top2-5* strain (Figure 5C). This finding further indicates that DNA at the *top2-5* anaphase bridges is broken at the time of PM abscission (Germann *et al.* 2011).

### Synthetic genetic array analysis confirms mitotic exit and cytokinesis as deleterious enhancers of transient Top2 inactivation

In order to weigh the overall negative contributions of mitotic exit and cytokinesis in each *top2-ts* allele, we carried out an SGA analysis with two collections of yeast mutants: the haploid gene deletion collection of nonessential genes (4322 knockout strains) and a collection of ts alleles for essential genes (1231 ts strains) (Tong *et al.* 2001; Giaever *et al.* 2002; Li *et al.* 2011). SGA allows screening for genetic interactions in *S. cerevisiae* by comparing the fitness of the different mutant combinations (*i.e.*, single mutants in the collections *vs.* double *top2-ts*/collection mutants). In addition, we decided to perform the SGA analysis in three different conditions that modify Top2 activity.

**Figure 6:**
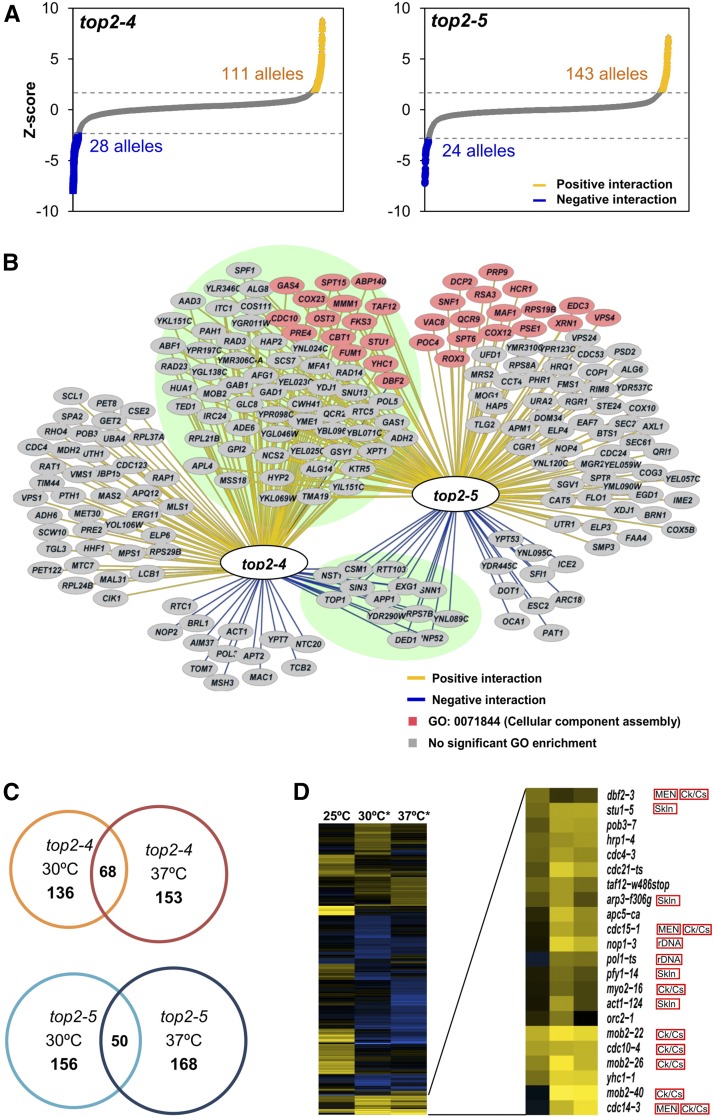
SGA analyses identify the MEN as deleterious enhancers of Top2 downregulation. (A) The z-scores of the genetic interactions detected at permissive temperature (steady growth at 25°) are plotted. The *top2-ts* arrays of mutants were compared with the *TOP2* arrays as a control. 5553 alleles were screened in total. Positive and negative genetic interactions that meet the cutoffs (>2 or < −2) are indicated. (B) Network of the genes that interact with *top2-4* and *top2-5*. Green background encircles the 84 shared genes between the two *top2-ts*. Pink nodes indicate the positive interaction between *top2-5* and genes involved in the aggregation and bonding of cellular components (GO: 0071844). (C) The numbers of genetic interactions identified during steady growth at 30° and after 6 hr incubation at 37° are shown. Circle intersections depict number of genetic interactions shared between both treatments. (D) Heat map of *top2-5* SGA scores (those with *P* < 0.05). Yellow, positive interactions; blue, negative interactions; black, no interaction. The 25° Column represents the z-score obtained comparing *top2-5 vs. TOP2* at 25°, whereas the 30° and 37° × 6 hr columns compare *top2-5* at these temperature regimes *vs. top2-5* at 25°. MEN, mitotic exit network; Ck/Cs, cytokinesis/cell separation; Skln, cytoskeleton; rDNA, ribosomal DNA metabolism.

In our first analysis, we grew all the strains constantly at the permissive temperature (25°), and compared the collection of *top2-4* and *top2-5* double mutants with the corresponding *TOP2* counterparts as references. Thermosensitive alleles are expected to have a mild reduced fitness at the permissive temperature and, in our case, we indeed detected genetic interactions at 25°, 139 in *top2-4* and 167 in *top2-5* (Figure 6A, Table S2, and Table S3 in File S1). Eighty-four interactions were shared between *top2-4* and *top2-5*, and these interacted with both alleles in a similar way, either positively or negatively (Figure 6B and Table S4 in File S1). Among them was *top1*Δ, which is well known to have synthetic sickness with *top2-ts* alleles (Kim and Wang 1989). However, most of the observed genetic interactions were positive, which is typically a sign of suppressive pathways.

Next, we sought to study the changes that increasing the temperature would produce in the observed genetic interactions. We opted for two different incubations: one set of arrays was grown at semipermissive temperature (30°) for 2 d, and another set was incubated at 37° for 6 hr, and then shifted to 25° to allow growth of survivors. This transient restrictive regime gives enough time to complete one cell cycle without Top2 on solid media (Figure 2A), and indeed allowed us to observe the *cdc15-2* protection of *top2-5* (Figure 4A). In both cases we used the *top2-ts* arrays that were grown at 25° as controls to compare colony sizes. Thus, we obtained a large number of new interactions that, unlike during constant growth at 25°, were mostly negative (Figure 6C, Table S5, and Table S6 in File S1). Ontological classification of significant interactions revealed common negative interactions at 37° × 6 hr with bioenergetics and autophagy (Table 1). This could be related to the heat shock treatment and putative roles of Top2 during chromosome reshaping and transcription reprogramming to cope with this stress (Pommier *et al.* 2016). Importantly, this classification also spotlighted a high number of positive interactions between *top2-5* grown at 30° and/or 37° × 6 hr and thermosensitive alleles related to mitotic progression, especially anaphase/telophase progression (Figure 6D and Table 1). Many of the genes belong to the MEN, including *CDC15*, whereas others are related to the cytoskeleton or rDNA metabolism, which are known to undergo important modifications during anaphase (Machín *et al.* 2016). To a lesser extent, *top2-4* was also enriched in mitotic division alleles when incubated at 37° × 6 hr, some of which overlap with those of *top2-5* (*e.g.*, *STU1*, *CDC10* and *MOB2*) (Table S5 and Table S6 in File S1).

**Table 1:** Significant biological processes that genetically interact with top2-ts in different downregulating regimes

SGA group*^a^*	Gene Ontology*^b^*	*P*-value*^c^*
*top2-4* – 30° (pos. int.)	Carbohydrate biosynthetic process (GO:0016051)	0.04323
		
*top2-4* – 37° x 6h (pos. int.)	APC-dependent ubiquitin-dependent protein process (GO:0031145) Cell division (GO:0051301)	0.00681 0.00706
		
*top2-4* – 37° x 6h (neg. int.)	Macroautophagy (GO:0034262)	0.03361
		
*top2-5* – 30° (pos. int.)	Macromolecular complex subunit organization (GO:0043933) Organelle assembly (GO:0070925)	0.00115 0.04773
		
*top2-5* – 37° x 6h (pos. int.)	Cell division (GO:0051301) Mitotic cell cycle process (GO:1903047) Mitotic cell cycle (GO:0000278) Mitotic nuclear division (GO:0007067) Mitotic cell cycle phase transition (GO:0044772) Cell cycle phase transition (GO:0044770) Cell separation after cytokinesis (GO:0000920) Nuclear division (GO:0000280) Regulation of cell division (GO:0051302)	1.49E-05 0.00017 0.00033 0.00035 0.0006 0.00068 0.02562 0.03884 0.04929
		
*top2-5* – 37° x 6h (neg. int.)	Macroautophagy (GO:0034262) Cellular respiration (GO:0045333) Oxidative phosphorylation (GO:0006119) Generation of precursor metabolites and energy (GO:0006091) Phosphorylation (GO:0016310) Autophagy (GO:0006914) Homoserine metabolic process (GO:0009092) Sister chromatid biorientation (GO:0031134) Energy derivation by oxidation of organic compounds (GO:0015980)	0.00048 0.00067 0.00238 0.00301 0.01924 0.02062 0.02391 0.02391 0.04017

a The corresponding *top2-ts* double mutant arrays were grown at 25° x 2 d, 30° x 2 d, and 6 hr x 37° + 25° x 2 d. The genetic positive interactions (pos. int.) and negative interactions (neg. int.) refer to the comparison between the downregulating temperature regimes and steady growth at 25°.

b Gene Ontology (GO) enrichment analyses were done using the Generic GO Term Finder (http://go.princeton.edu/cgi-bin/GOTermFinder). GO terms that contained > 1500 genes were discarded, as they are usually too general. Similarly, redundant GO terms with < 10 genes were discarded.

c *P*-values were computed using a hypergeometric distribution, and adjusted with the Bonferroni correction.

## Discussion

In this work we have presented single-cell biology studies and large-scale genetic interaction data that strongly support that the survival of a yeast cell transiently depleted from Top2 is negatively correlated with the commitment to execute cytokinesis at the end of mitosis. To a great degree, our data confirm results by others who have previously explored such a possibility (Baxter and Diffley 2008). Importantly, we have classified the different aberrant nuclear morphologies seen in *top2-ts* anaphases and correlated them with the degree of completion of cytokinesis.

In addition, we have found many positive genetic interactions between *top2-ts* and alleles or mutations that delay different stages of the cell cycle (Figure 6, Table 1, Table S2, Table S3, Table S4, Table S5, and Table S6 in File S1). We reason that such positive interactions, measured as improved fitness on a SGA, are better explained by: (i) fewer cells with CABs reaching the point-of-no-return (*i.e.*, cytokinesis) during the transient 37° × 6 hr shift; and (ii) the additional cell cycle delay allowing the reduced Top2 activity to end up resolving catenations, especially in the case of steady incubations at 25 and 30°. Again, these positive interactions support previous works that claimed that blocking the cell cycle in G1 or G2/M protects against transient Top2 depletion (Holm *et al.* 1985; Thomas *et al.* 1991).

Finally, our work also put forward some intriguing questions about why downregulation of Cdc14, the trigger of exit from mitosis, does not protect against Top2 deficiency. Last but not least, we uncovered surprising differences between the two *top2-ts* alleles used in this work, *top2-4* and *top2-5*.

### On the nature and fate of the anaphase bridges that result from depleting Top2

We started this work by revisiting earlier works from Botstein’s and Sternglanz’s laboratories on yeast cells cycling without Top2 activity (DiNardo *et al.* 1984; Holm *et al.* 1985). These studies reported that *S. cerevisiae* had no terminal phenotype at the *top2-ts* restrictive temperature. Rather, a mixture of unbudded and large-budded (dumbbell) cells was the final outcome of Top2 inactivation. In many dumbbell cells, failure in chromosome segregation was evident due to the presence of DAPI-stained anaphase bridges. They assumed that there were two classes of cells coming from a *top2-ts* G1-synchronized culture: those which get stuck as dumbbells with major defects in nuclear segregation, and those which complete cytokinesis and cell separation. In light of the unbudded progeny they observed, they concluded that the latter had gone through a devastating mitotic catastrophe. In general, our data fully support this conclusion (profiles of cell morphologies in Figure 1 and Figure 2). The most shocking difference is the relatively low percentage of visible anaphase bridges, which we here refer to as CABs since we used histone-labeled DNA (H2A-GFP). Of note, we also observed a small fraction of anaphase bridges when staining with either DAPI or Hoechst and, in addition, H2A-GFP and DAPI signals perfectly colocalized (Figure 2B). Instead of CABs, we often distinguished very close split nuclear masses just across the neck (SD-binucleated). The distance between the split signals was normally <1 μm. It is likely that this phenotype has been referred to as “anaphase bridge” in previous works. Notably, a similar phenotype was described before for a strain that depletes Top2 through a degron system and referred to as “cut” phenotype (Baxter and Diffley 2008). Besides, a similar terminal phenotype occurs in mouse topo IIα^−/−^ embryos and in a significant fraction (∼1/3) of epithelial cells treated with Top2 catalytic inhibitors (Wheatley *et al.* 1998; Akimitsu *et al.* 2003). Outstandingly, we have been able to correlate this SD-binucleated phenotype to ingression of the cleavage furrow at the cytokinetic plate. On the one hand, we observed that CABs were only visible when ingression was absent (Figure 3B). On the contrary, SD-binucleated required either full contraction or resolution of the PM at the neck (abscission). On the other hand, when we blocked MEN in *top2-ts* by depleting Cdc15, and hence we also blocked cleavage furrow ingression, the SD-binucleated morphology was absent and a CAB was seen instead (Figure 4C and Figure S4 in File S1). As mentioned already, the large-scale screen for genetic interactions also supports that commitment to execute cytokinesis is deleterious for *top2-ts* (Figure 6, Table 1, Table S2, Table S3, Table S4, Table S5, and Table S6 in File S1).

An intriguing question is what happens to the CAB during PM ingression and abscission. Two alternative hypotheses were possible: (i) the CAB is broken through cytokinesis, or (ii) the CAB becomes a histone- and DAPI-invisible UFB. Notably, a recent report has shown that thin channels of nuclear material are formed between the mother and the daughter cells when Top2 is depleted (Amaral *et al.* 2016). These channels go through cells that appear to have completed PM abscission, are fully surrounded by septum, and might be wide enough to accommodate UFBs. These channels might also explain why we observed PM abscission in many *top2-ts* dumbbells but were unable to split them upon zymolyase treatment (Figure 3, A and B). We have tried to address the fate of CABs during PM ingression by triple-labeling the PM, the CAB (H2A-GFP), and the UFB (Dpb11-RFP). Our data do support that most CABs (and UFBs) are broken apart at the time of PM abscission, (Figure 5). Thus, there was no change in the CAB/UFB ratio during the drop of anaphase bridges by PM abscission in *top2-5*. In addition, Dpb11 relocalized to foci which, together with the formation of Rad52 foci (Figure 1A), strongly points toward DSBs taking place in *top2-5* late anaphase. Since PM abscission, CAB/UFB disappearance, and Dpb11 foci accumulation could all be prevented in the *top2-5 cdc15-2* double mutant, we favor the hypothesis that all kinds of anaphase bridges are broken as a consequence of cytokinesis. Nevertheless, the alternative hypotheses of (i) Dpb11 relocalizing from UFBs to DNA repair foci upon CAB breakage or (ii) the UFB becoming too narrow for the current limits of fluorescence microscopy cannot be ruled out entirely and deserve further investigation in the future, especially in the light of the aforementioned channels.

**Figure 7:**
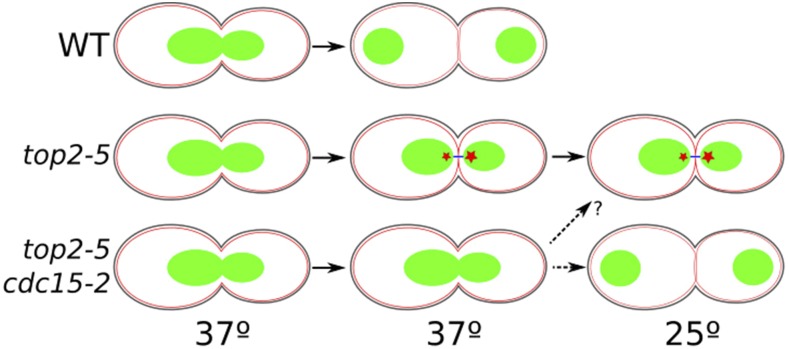
Summary and model of anaphase progression in *top2-5* and *top2-5 cdc15-2* mutants. Cells with wild-type (WT, upper schematic) Top2 enter anaphase and quickly resolve catenations to yield LD-binucleated cells. Temperature-sensitive *top2-5* (middle schematic) also enters anaphase on schedule but fails to resolve catenations, yielding a chromatin anaphase bridge (CAB). CABs cover a short distance across the neck in many instances (SD-phenotype). Importantly, cytokinetic furrow ingression is not blocked and results in CABs being visibly severed into binucleated morphologies (Figure 1, Figure 2, Figure 3, Figure 4, and Figure 5). Two scenarios are possible: DNA is actually severed by cytokinesis, or the CAB is turned into a sort of Dpb11-free ultrafine bridge (blue line) (Figure 5). In any case, DNA damage arises and is sensed in the progeny (red stars). Damage might be worse in the daughter cells since ∼20% of the mothers can rebud at least once (Figure 2). Blocking cytokinesis in *top2-5 cdc15-2* (lower schematic) stabilizes CABs. Resuming Top2 function after anaphase in *top2-5* (shift to 25°) yields an unviable progeny. Resuming Top2 and cytokinesis (Cdc15) at the same time gives a window of opportunity to recover and may yield at least one viable cell (dashed arrows). Green objects depict the nuclear masses (histone-labeled DNA); black lines on the cell surface depict cell wall; red thin lines depict the PM.

Altogether, we propose a model where cells depleted from Top2 always form CABs at anaphase (Figure 7). These CABs can be massive and might often prevent the mitotic spindle from pulling the segregating sister chromatids away from each other, hence the short distances that separate the splitting masses across the bud neck. Alternative possibilities for such short distances are feasible but unlikely; *e.g.*, Top2 is known to play no roles during mitotic spindle enlargement (Andrews *et al.* 2006). Importantly, these CABs do not prevent cleavage furrow ingression, which quickly changes them into SD-binucleated (Figure 3B). Remarkably, cells depleted from the condensin subunit Brn1, which are thought to give rise to *top2*-equivalent anaphase bridges, do not delay execution of cytokinesis either (Cuylen *et al.* 2013), and only a short delay of 15 min was seen for karyokinesis in anaphase bridges restricted to the rDNA (Quevedo *et al.* 2012). What happens after full membrane ingression is less clear. However, DNA damage seems apparent (Figure 1 and Figure 5), suggesting that DNA is severed as reported previously (Holm *et al.* 1989; Baxter and Diffley 2008). Again condensin depletion leads to similar results (Cuylen *et al.* 2013). Strong support for this model was obtained when we blocked MEN and cytokinesis (*i.e.*, *cdc15-2*) in *top2-5* strains. Thus, CABs were stabilized in the *top2-5 cdc15-2* double mutant and no signs of DNA damage were detected (Figure 1 and Figure 5). Strikingly, though, even complete block of MEN yielded only partial recovery when Top2-5 was reactivated (Figure 4A). In our study, this recovery was slightly smaller than the one previously reported (Baxter and Diffley 2008), likely due to the fact that we incubated the cells at 37° for a longer period. It is probable that the partial recovery relates to cells restoring Top2 and Cdc15 functions at the same time after the 37–25° shift, although the possibility of CABs becoming irreversible upon a long absence of Top2 should not be ruled out completely. Indeed, we have shown before that rDNA bridges in *cdc14-1* eventually become challenging to resolve (Machín *et al.* 2006; Quevedo *et al.* 2012).

### On the putative synergistic role of Cdc14 in anaphase bridge resolution through the FEAR network

Top2 is not the only universal player needed to avoid the occurrence of CABs during the mitotic cell division. The condensin complex also plays a key role in preventing CABs throughout all life kingdoms (Kalitsis *et al.* 2017). Previous works have placed condensin and Top2 within the same pathway to accurately remove sister chromatid catenations in *S. cerevisiae* (Baxter *et al.* 2011; Charbin *et al.* 2014). The master mitotic phosphatase Cdc14 controls resolution of sister chromatids by acting on overall transcription and thus favoring condensin localization onto DNA, conditions that are especially critical for the highly transcribed rDNA array (Machín *et al.* 2016). This Cdc14 control over condensin takes place in early anaphase and is possible because Cdc14 is transiently activated there through the FEAR network. Cdc14 also plays an essential role for exit from mitosis at late anaphase through its second activation by MEN. The kinase Cdc15 is critical for Cdc14 activation by MEN but not by the FEAR network (Stegmeier and Amon 2004). In this work, we have seen two important differences when the *top2-5* allele was combined with ts mutants for the *CDC14* and *CDC15* genes. First, *top2-5 cdc15-2* could partly rescue the reduced fitness of *top2-5*, whereas *top2-5 cdc14-1* did not (Figure 4, A and B). This was confirmed in the SGA analysis, where *cdc15-1* (*cdc15-2* was not present) also alleviated *top2-5*; whereas two out of the three included *cdc14-ts* alleles were neutral (*cdc14-1* and *cdc14-2*; just *cdc14-3* emerged as a suppressor). Second, *top2-5 cdc15-2* led to CABs that resembled the morphology of the SD- and LD-binucleated nuclear masses in *top2-5*; *i.e.*, addition of a bridge to these morphologies was the only difference (Figure 4C). Nevertheless, *top2-5 cdc14-1* gave a missegregation pattern that was clearly worse than either ts allele alone. In most cases the nuclear mass failed to split entirely, yet it localized within the daughter cell (Figure 4D). This phenotype is reminiscent of cells depleted of separase, the protease that breaks sister chromatid proteinaceous cohesion at the anaphase onset (McGrew *et al.* 1992). Taking into account that *cdc14-1* strains are also known to mistakenly segregate the nucleus to the daughter cell (Ross and Cohen-Fix 2004), the synergistic nuclear segregation defect in *top2-5 cdc14-1* may be due to the lack of a FEAR-dependent spindle pulling force back toward the mother. The alternative hypothesis is that Top2 and Cdc14 actually work in parallel pathways. This hypothesis would thus put forward that either condensin has Top2-independent roles or, alternatively, Cdc14 controls condensin-independent processes important for sister chromatid resolution. In support of the latter, Cdc14 depletion also results in an enrichment of regions with unfinished replication, particularly in the rDNA, as well as sister chromatids still connected through recombination intermediates (Dulev *et al.* 2009; García-Luis *et al.* 2014). However, similar problems have been described in *top2* mutants as well (Baxter and Diffley 2008; Fachinetti *et al.* 2010). Finally, it is worth mentioning that the highly asymmetric segregation of the nucleus we observed in *top2-5 cdc14-1* is the hallmark of mammal epithelial cells treated with Top2 catalytic inhibitors (Gorbsky 1994; Wheatley *et al.* 1998).

### On the similarities and differences between top2-4 and top2-5

Temperature-sensitive alleles for *TOP2* were originally thought to be equivalent. In the first screenings for such *top2-ts* alleles, just one was normally selected and deeply studied, assuming that the other *top2-ts* were identical. That was the case for *top2-4*, which has been widely used since and founded most of the knowledge we have about Top2 functions in yeast. Nevertheless, independent isolation of *top2-ts* from different labs showed later that *top2-ts* could carry intrinsic and variable properties in terms of cell cycle progression, commitment to enter anaphase, etc. (Andrews *et al.* 2006). One of the first surprising findings was that there was a difference in terms of resistance to the clinically used Top2 poisons within the same *top2-ts* group where *top2-4* was isolated. Thus, *top2-5* proved to be resistant to poisons even at permissive temperature (Jannatipour *et al.* 1993). This fact led us to include coisogenic *top2-4* and *top2-5* strains in this work. Surprisingly, we found marked differences between them. In general, both *top2-ts* alleles shared many similarities: (i) they failed to arrest in G2/M; (ii) they formed short-lived CABs, which could be stabilized by depleting Cdc15; (iii) they had DNA damage coinciding with anaphase progression; (iv) they led to two classes of split nuclear masses in anaphase (SD- and LD-binucleated); and (v) the immediate progeny often failed to bud again, and when they did, budding was restricted to the mother. The differences were more related to the timing of the cell cycle events and relative proportions of several phenotypes (Figure 1). It is important to highlight that these differences were more obvious in time course experiments carried out in liquid cultures than when we filmed single cells on agarose patches (Figure 2). In the time course experiments, *top2-5* progressed through the cell cycle faster than *top2-4*. It also became stalled as dumbbells less frequently than *top2-4*. Likewise, SD-binucleated occurred less frequently in *top2-5*. Since *top2-5* had a quicker G1-S transition in cultures relative to cells filmed on agarose patches, the most trivial explanation for these differences lay in this G1-S transition, making later phenotypes appear earlier and evolve into others quicker. As for the reason behind this difference, we can hypothesize about two origins. On the one hand, the difference may relate to the genetic history of these strains through the numerous passes over the years. Taking into account that it has been shown that Top2-5 only keeps 33% of normal Top2 activity at 25° (Jannatipour *et al.* 1993), we believe that the *top2-ts* strains might be genetically unstable at 25°, which would in turn boost the probability of *top2-4* and *top2-5* being genetically different despite their coisogenic origins. On the other hand, there might be an intrinsic difference between the *top2-ts* alleles. In support of this second hypothesis we have the fact that the *top2-5* allele still gathered more genetic interactions than *top2-4* when transferred to the SGA background. It is difficult to speculate on the causes of such an intrinsic difference. There is only a missense mutation in the protein encoded by *top2-4*, a proline-to-glutamine change at position 820 (P820G), whereas Top2-5 has three in a cluster: R883P, R885I, and M887I (Thomas *et al.* 1991; Jannatipour *et al.* 1993). In both cases, mutations lay in the gyrase A motif near the catalytic center of the enzyme (Y782). Although it is plausible that the three mutations of *top2-5* render the enzyme less active than Top2-4, more work is needed to conclusively explain intrinsic differences upon the 37° shift. Lastly, an interesting possibility is that differences may correlate with the poison-resistant nature of Top2-5. This latter scenario would have important health-related implications and, therefore, it is worth testing in future works.

### Conclusions and perspectives

In this work, we have characterized the consequences of depleting yeast cells from Top2 using two *top2-ts* alleles that differ in their resistance to chemotherapeutic Top2 poisons. As previously reported, the first cell cycle takes place with normal kinetics until anaphase, when *top2-ts* forms anaphase bridges. We have shown that these bridges are quickly split apart by PM abscission and can be maintained if mitotic exit is blocked. Finally we provide genetic evidence that stabilization of these bridges improve survivability upon transient Top2 inactivation. Since transient inactivation of targets is of the outmost importance to predict cell response to pharmacological drugs and also uncover adjuvant treatments, our results point toward upregulation of mitotic exit as a putative target to synergistically promote cell death upon Top2 downregulation/mutation in cancer cells.

## Supplementary Material

Supplemental material is available online at www.g3journal.org/lookup/suppl/doi:10.1534/g3.117.300104/-/DC1.
