# Supplementary material for: Genome-Scale Genetic Interactions and Cell Imaging Confirm Cytokinesis as Deleterious to Transient Topoisomerase II Deficiency in Saccharomyces cerevisiae
Source: G3 (Bethesda). 2017 Aug 23;7(10):3379–91. doi: 10.1534/g3.117.300104 (PMC5633387; doi:10.1534/g3.117.300104)
Supplement: Supplementary file 1 [file 3379FileS1.pdf]

Genome-scale genetic interactions and cell imaging confirm cytokinesis as deleterious to transient Topoisomerase II deficiency in *Saccharomyces cerevisiae*.

Cristina Ramos-Pérez[*,†,1], Jessel Ayra-Plasencia[*,†], Emiliano Matos-Perdomo[*,†], Michael Lisby[‡], Grant W Brown[§] and Félix Machín[*,2]

[*] Unidad de Investigación, Hospital Universitario Nuestra Señora de Candelaria, 38010, Santa Cruz de Tenerife, Spain.

[†] Universidad de la Laguna, 38200, San Cristóbal de La Laguna, Santa Cruz de Tenerife, Spain.

[‡] Department of Biology, University of Copenhagen, DK-2200, Copenhagen, Denmark.

[§] Department of Biochemistry and Donnelly Centre, University of Toronto, M5S3E1, Toronto, Ontario, Canada.

[1] Present address: Department of Biochemistry and Donnelly Centre, University of Toronto, M5S3E1, Toronto, Ontario, Canada.

[2] Corresponding author:

Félix Machín. Unidad de Investigación, Hospital Universitario Nuestra Señora de la Candelaria, Ctra del Rosario 145, 38010 Santa Cruz de Tenerife, Spain. Tel: +34922602951. E-mail: fmachin@funcanis.es

SUPPLEMENTAL MATERIAL AND METHODS.

Construction of yEmRFP-2xPH

First, a BamHI/SalI fragment of vector pML105 (Germann et al. 2014) containing PRC1p-CFP-2xPH was subcloned into the BamHI/SalI-digested *HIS3* vector pML104 (Germann et al. 2014) to generate pML106. Next, CFP was replaced with yEmRFP in vector pML106 by fusing a PRC1 promoter PCR fragment generated with BamHI-adapted primers PRC1-F and PRC1-yEmRFP-R from template pRS426GFP-2×PH(PLCδ) (Stefan et al. 2002) to a yEmRFP PCR fragment generated with BspEI-adapted primers cherry.Fw and yEmRPFend-BspEI-R from template pNEB30 (Silva et al. 2012). The PCR fusion product was digested with BamHI and BspEI and cloned into BamHI/BspEI-linearized pML106 to produce plasmid pML111. The pML111 plasmid was partially digested with BsmI and transformed into strain ML8-9A for integration at the *his3-11,15* locus producing strain ML704.

Primers related to this construction were (5' to 3'):

PRC1-F	GTGGATCCTTCTGCACAAGAAG
PRC1-yEmRFP-R	CTTCTTCACCTTTTGAAACCATAGCGTATGTATACTTTAAG
cherry.Fw	ATGGTTTCAAAAGGTGAAGAAG
yEmRFPend-BspEI-R	GAGTCCGGATTTATATAATTCATCCATACCACC

References:

Germann SM, Schramke V, Pedersen RT, Gallina I, Eckert-Boulet N, Oestergaard VH, Lisby M. 2014. TopBP1/Dpb11 binds DNA anaphase bridges to prevent genome instability. *J Cell Biol* **204**: 45-59.

Silva S, Gallina I, Eckert-Boulet N, Lisby M. 2012. Live Cell Microscopy of DNA Damage Response in *Saccharomyces cerevisiae. Methods Mol Biol* **920**: 433-443.

Stefan CJ, Audhya A, Emr SD. 2002. The yeast synaptojanin-like proteins control the cellular distribution of phosphatidylinositol (4,5)-bisphosphate. *Mol Biol Cell* **13**: 542-557.

SUPPLEMENTAL FIGURES.

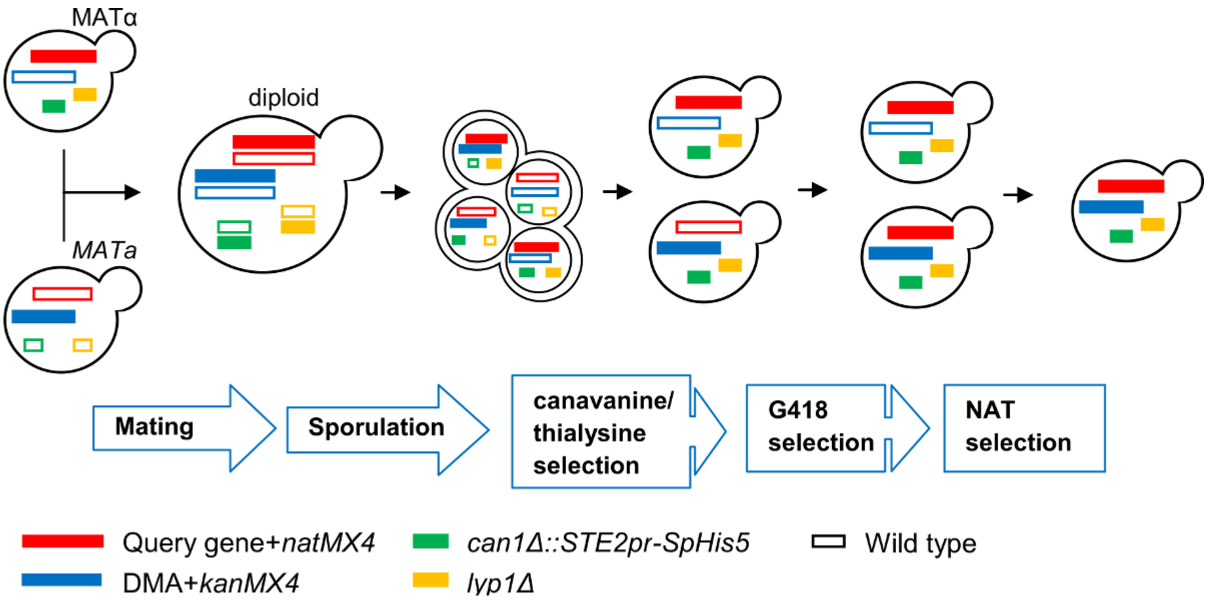

Figure S1. Schematic of the generation of strain arrays for SGA analysis. This technique is designed to detect genetic interactions by creating a subset of strains that have one query mutated gene in common and an array of other known mutations or deletions in their genome. We used the ts alleles *top2-4* and *top2-5* as our query genes, and scanned for genetic interactions with an array of mutants with deletions for 4322 non-essential genes and 1231 thermosensitive essential genes. We first replaced the *TOP2* locus in a haploid *MAT***α** strain (strain Y7092) with our query *top2-ts* alleles attached to the selection marker *natMX4* (resistance to nourseothricin -NAT-). For consistency, we also attached *natMX4* to the wild type *TOP2* locus, resulting in three Y7092 derivatives. The target Y7092 strain also carries other dominant selection markers such as *can1*Δ and *lyp1*Δ, which confer resistance to canavanine and thialysine, respectively. In addition, the deletion of *can1*Δ is linked to the construct *STE2p-SpHis5*. *STE2p* is a promoter that is only active in *MAT***a** haploid cells; allowing them to grow in media lacking histidine. The new *TOP2:natMX4*, *top2-4:natMX4* and *top2-5:natMX4* strains were then mated with the *MAT***a** deletion mutant array (DMA) and the ts mutant array (TSv6), which bear the *kanMX4* marker (resistance to geneticin -G418-) at the mutated loci. The result of this mating is a diploid with heterozygosity for all the markers. To select only the diploids and discard the cells that did not mate, the arrays were grown on plates containing both NAT and G418. After this, the diploids were plated onto sporulation media for 5 - 7 days. The plates were then replicated onto SD/MSG -his -arg -lys media with canavanine and thialysine in order to select for spores carrying the *MAT***a** *can1*Δ *lyp1*Δ genotype. The lack of histidine only allows the growth of *MAT***a** haploid (spores) cells carrying the *STE2pr-SpHis5* construct, whereas canavanine and thialysine kill all the cells carrying the WT alleles *CAN1* and *LYP1*, including all diploid. Next, we replicated the array onto the same media but adding G418, in order to select cells that carried the deletion mutation of the DMA or ts collection. Finally, we made a last selection onto the same media, this time adding also NAT, to select only the mutants that bear our query *TOP2:natMX4*, *top2-4:natMX4* and *top2-5:natMX4* alleles. Once the *TOP2*, *top2-4* and *top2-5* arrays were constructed they were replicated onto plates with the same medium used in the last selection step to maintain the selective pressure, and they were exposed to the different temperature regimes described in the Results section. The aforementioned genes are indicated with boxes with different sizes and colour lines. The presence of a modified allele is indicated by a filled box, whereas the WT allele is an empty box. Details on the different media compositions can be found at Tong, A. & Boone, C. Yeast Gene Anal. - Second Ed. 36, 369–707 (2007).

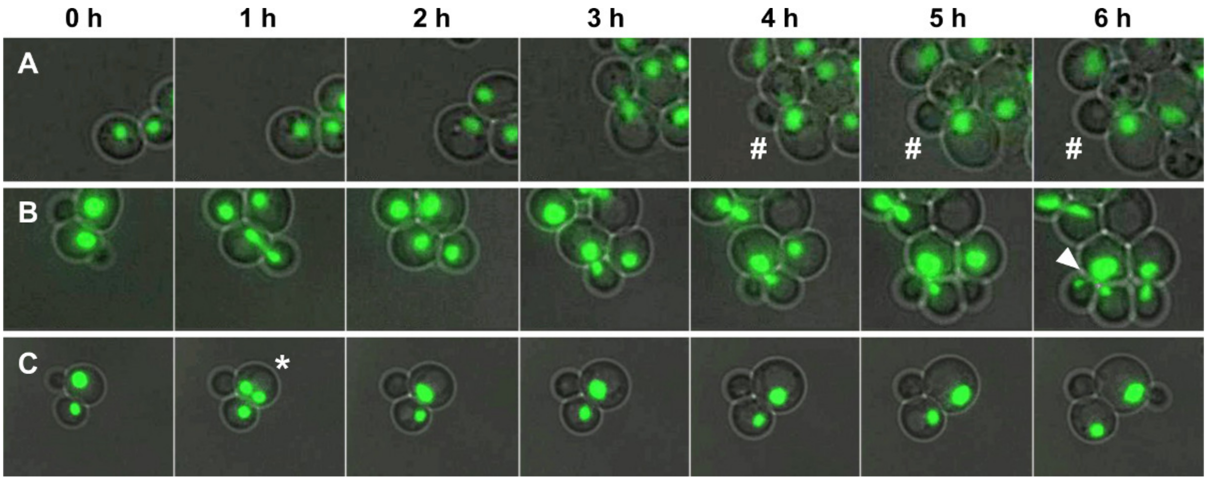

Figure S2. Examples of uncoupling between nuclear division and cell cycle in *top2-ts* mutants. *HTA2-GFP top2-ts* cells were filmed as in Figure 2A. **A.** A cell in which a new bud (#) comes out from the mother before it had finished segregating the DNA with the first bud (observed in two *top2-4* cells). **B.** An example of the formation of a double CAB (arrowhead) that connects the mother cells with its two daughters (observed in one *top2-5* cell). **C.** A nucleus trying to divide inside the mother cell, resulting in a temporarily binucleated single cell (*) that then fuses the split nuclei back into one single nuclear mass (observed in one *top2-4* cell). The total number of cells filmed was 239 and include both *top2-ts* mutants as well as cells which were in either G1 or S phase (small bud) at the time of the temperature shift.

top2-4
cdc15-aid
H2A2-GFP

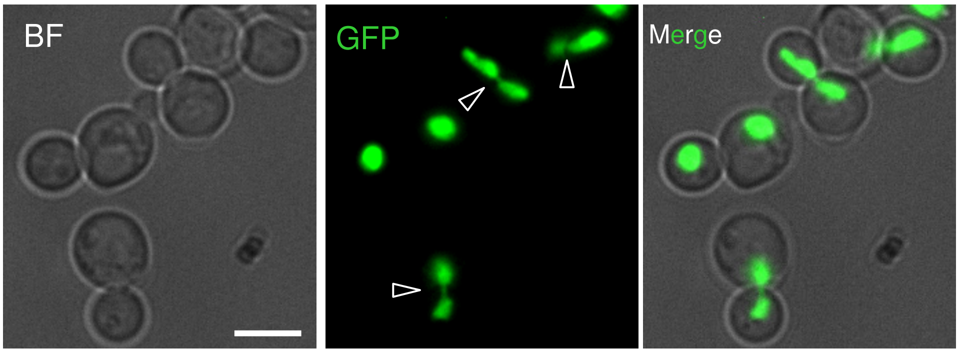

Figure S3. Chromatin anaphase bridges are stabilized by blocking Mitotic exit in *top2-4*. The *top2-4 cdc15-aid HTA2-GFP* strain was synchronized in G1 at 25º and then released at 37º in the presence of 1 mM of the auxin indolacetic acid (IAA) for 3 h. Scale bars correspond to 5 µm. BF, bright field. Hollow arrowheads point to examples of CABs. All arrowheads point exactly at the bud neck.

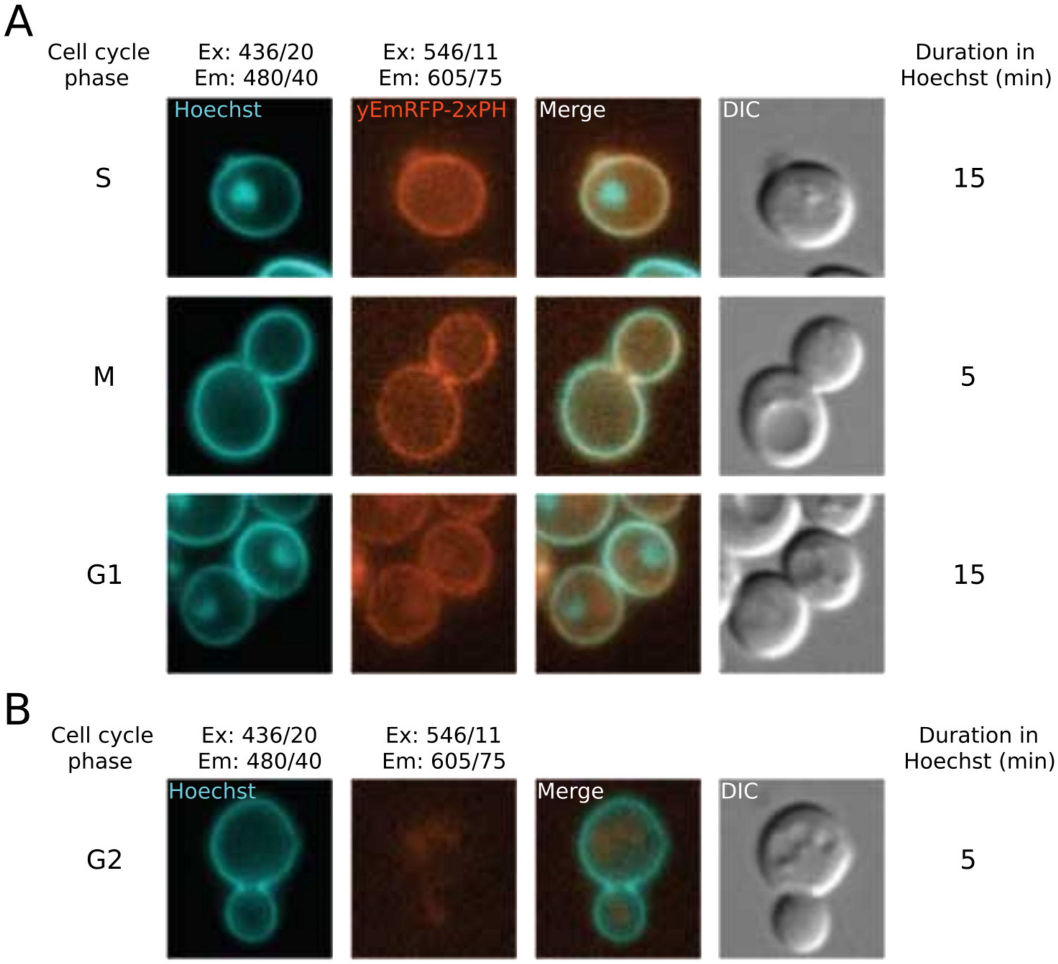

Figure S4. Validation of Hoechst staining as a marker for the plasma membrane. A. Hoechst colocalizes with the plasma membrane marker 2xPH. Cells expressing the plasma membrane marker yEmRFP-2xPH (ML704) were grown to exponential phase (OD = 0.2) in SC+Ade medium and stained with 5 µg/ml Hoechst 33258 for 5 min or 15 min as indicated before mounting on a microscope slide in fresh SC+Ade medium without Hoechst. Images show representative cells in S, M and G1 (post-abscission) phase of the cell cycle. **B.** Staining of control cells. Wild-type cells without the yEmRFP-2xPH marker (ML8-9A) were stained with 5 µg/ml Hoechst 33258 for 5 min before mounting on a microscope slide in fresh SC+Ade medium without Hoechst to confirm that Hoechst does not bleed-through into the RFP channel. DIC, differential interference contrast.

SUPPLEMENTAL TABLES.

Table S1. Strains used in this work.

Strain name	Relevant genotype[a]	Origin
CH325	(S288C) *MAT***a** *ura3-52 his4-539am lys2-801am SUC2+ top2-4*	D. Botstein
CH326	(S288C) *MAT***a** *ura3-52 his4-539am lys2-801am SUC2+ top2-5*	D. Botstein
CH335	(S288C) *MAT***a** *ura3-52 his4-539am lys2-801am SUC2+ TOP2*	D. Botstein
FM1386	CH326; *H2A2(YBL003c):GFP:BleMX*; *Δbar1::URA3*	This work
FM1387	CH325; *H2A2(YBL003c):GFP:BleMX*; *Δbar1::URA3*	This work
FM1419	CH335; *H2A2(YBL003c):GFP:BleMX*; *Δbar1::URA3*	This work
FM1423	FM1387; *RAD52:RedStar2:NatMX*	This work
FM1437	FM1419; *RAD52:RedStar2:NatMX*	This work
FM1457	FM1386; *RAD52:RedStar2:NatMX*	This work
FM2021	FM1386; *cdc15-2:9myc:Hph*	This work
FM2086	FM1419; *cdc15-2:9myc:Hph*	This work
FM2105	CH326; [2·PH-GFP:URA3]	This work
FM2153	FM1386; *cdc14-1:9myc:Hph*	This work
FM2152	FM1419; *cdc14-1:9myc:Hph*	This work
FM2147	CH325; *H2A2(YBL003c):GFP:BleMX*; ura3-52:ADH1:OsTIR1:9Myc:URA3; *cdc15:AID*:Hph*	This work[b]
ML8-9A	(W303) *MAT***a** *ADE2 can1-100 ura3-1 his3-11,15 leu2-3,112 trp1-1 LYS2 RAD5*	M. Lisby
ML704	ML8-9A; *his3-11,15:yEmRFP-2xPH:HIS3*	This work
ML1009	FM1386; *tTA(tetR-VP16)-tetO2-DPB11-yEmRFP::KanMX*	This work[c]
ML1010	FM2021; *tTA(tetR-VP16)-tetO2-DPB11-yEmRFP::KanMX*	This work[c]
ML1022	FM2086; *tTA(tetR-VP16)-tetO2-DPB11-yEmRFP::KanMX*	This work[c]
Y7092	(S288C) *MAT***α** *can1Δ::STE2pr-Sphis5 lyp1Δ his3Δ1 leu2Δ0 ura3Δ0 met15Δ0*	C. Boone

FM2181	Y7092; *TOP2:natMX*	This work[d]
FM2211	Y7092; *top2-4:natMX*	This work[d]
FM2241	Y7092; *top2-5:natMX*	This work[d]

[a] Semicolons separate independent transformation events during strain construction. Intermediate strains are omitted. Brackets indicate the relevant genotype is in a plasmid.

[b] This strain is *BAR1*. *AID** stands for auxin-inducible degron system (asterisk denotes it carries the minimum effective sequence as reported in Morawska, M. & Ulrich, H. D. *Yeast* **30,** 341–51 (2013)). The Cdc15 depletion was checked by absence of growth on a 1 mM auxin (indolacetic acid, IAA) YPD plate and full telophase arrest at 25ºC after 3h with 1 mM IAA (data not shown).

[c] The *tTA(tetR-VP16)-tetO2-DPB11-yEmRFP::KanMX* cassette was PCR amplified an integrated at the *DPB11* locus.

[d] These strains are the basis for the corresponding *MAT***a** double mutants strain arrays (5,553 strains per *top2* allele; 16,659 in total), whose individual genotypes are omitted for the sake of space.

Table S2. Genes that interact exclusively with *top2-4* at 25ºC.

**Positive interaction**[a]	*ACA1, ADH6, AFR1, APQ12, AVT5, BLI1, BSP1, CCC2, CDC123, CDC19, CDC26, CDC37, CIK1, CSE2, CTH1, DBF4, DEP1, DON1, DUS3, ECM10, ECM23, EFT1, ELO1, ELP6, EMP46, ENT5, ERG11, ERG3, ESA1, FLD1, FMP41, FRE1, GCV1, GET2, GIR2, GRE2, HHF1, HKR1, INM2, IPT1, IST1, LCB1, LEA1, MAL31, MAS2, MBA1, MDH1, MDH2, MET30, MIC23, MLS1, MNS1, MPS1, MSB2, MSC7, MTC7, NAT5, NIT3, NPA3, NSE3, PBI2, PCF11, PCL9, PET122, PET8, PIN4, PPH3, PRE2, PTH1, PUT2, RAP1, RAT1, RAX2, RBD2, REX2, RHO4, RNH203, RPL24B, RPL37A, RPS29B, RPS6B, RSF1, SAS4, SCL1, SCW10, SCW11, SDH1, SGF73, SIF2, SIP3, SIP4, SKI2, SLI1, SLX4, SPA2, STU2, SWD3, SWT1, TIM44, TRK2, TVP23, UBA4, UBP15, UFD2, UTH1, VMS1, VPS1, YBL059W, YDR018C, YDR133C, YDR391C, YGL015C, YGL152C, YGL159W, YGR012W, YGR266W, YHR022C, YHR138C, YJL150W, YKL053W, YKL077W, YKR051W, YLR063W, YMR010W, YNL134C, YOL019W, YOL106W, YOR059C, YOR325W, YPL062W, YRB30*
**Negative interaction**[a]	*AAD3, ABP140, ACT1, AIM18, AIM24, AIM26, AIM37, ALP1, AMD1, APS1, APS2, APT2, ARO80, ATG1, ATO2, ATP18, AVL9, BOP2, BRL1, CBR1, CDC21, CIC1, CKB1, COA1, COX23, CPR7, CWH41, CWH43, DEG1, EAR1, ECM19, ERC1, ERG5, EXG1, FMP25, FMP46, GAL83, GDE1, GRX6, HAP3, HDA1, HIM1, HMI1, IME2, KAR4, LCB1, LSM6, MAC1, MAM3, MCK1, MDM38, MET30, MGS1, MHT1, MIG1, MKC7, MKS1, MNL2, MRPL10, MSH3, MTC3, NHP10, NOP2, NSE4, NSE5, NTC20, OPI1, ORM2, OSW2, PAN2, PCL1, PDC1, PET122, PET20, PEX13, PHB2, PHD1, PIN2, PMD1, PML1, POB3, POL3, POL32, PRM2, PTM1, PUN1, RFX1, RHO4, RNH1, RPA14, RPA34, RPL14A, RPL34B, RPL40B, RPL8A, RPS11A, RPS25B, RPS29B, RPS4B, RRT12, RTC1, RTC4, RUD3, SAM2, SCY1, SDC1, SEC23, SEC26, SHH4, SHO1, SKG3, SLA1, SLM4, SNA2, SPA2, SSO2, STF1, STP3, STP4, SUA7, SVL3, SWA2, SWC7, SYC1, SYF1, TCB2, TFS1, THI7, THI73, TOM7, TOS6, TRM1, UBC12, UBP6, UBP8, UGA2, UPS2, URC2, UTH1, VAM7, VPS1, VPS51, VRP1, YAR1, YBR287W, YCF1, YCK3, YCT1, YDJ1, YDL119C, YDR161W, YEL059W, YGR250C, YGR272C, YHR140W, YJL021C, YKR104W, YLR108C, YLR152C, YLR236C, YLR241W, YLR269C, YLR391W, YLR444C, YML035C-A, YNL171C, YNL195C, YNR040W, YOL029C, YOL106W, YOR062C, YPL034W, YPL150W, YPR084W, YPR197C, YPT7*

[a] normalized growth of double *top2-ts/collection* mutants are compared to that of single collection mutants, both grown at 25ºC.

Table S3. Genes that interact exclusively with *top2-5* at 25ºC.

**Positive interaction**[a]	*ABZ2, ACE2, ACT1, AIM5, AIM7, ALD4, ALG6, ALT1, APJ1, APM1, ATG2, ATX1, AVT4, AXL1, BRN1, BTS1, CAT5, CCT4, CCW12, CDC14, CDC15, CDC24, CDC53, CGI121, CGR1, CLA4, CLB1, COG3, COP1, COX10, COX12, COX5B, CPR5, CSF1, CTS1, DAN1, DBF2, DCP2, DLD1, DOM34, EAF7, EAR1, EDC3, EGD1, ELM1, ELP3, ELP4, EOS1, FAA4, FLO1, FMS1, GAL83, HAP5, HCH1, HCR1, HDA1, HNT2, HOM3, HRP1, HRQ1, IME2, IRC6, IRC8, KNS1, LAT1, LCB2, LSC2, MAF1, MCH1, MCK1, MCX1, MDJ2, MEP1, MGR2, MOG1, MPH1, MRP8, MRS2, MYO2, NOP1, NOP4, OAF3, OAR1, OCA4, OCA6, ORC2, PAC11, PAM17, PCK1, PHO90, PHO91, PHR1, PLP1, PMP3, POC4, POL1, PRC1, PRP9, PSD2, PSE1, PUF3, PUT3, QCR9, QRI1, RAD16, RCY1, RGR1, RIM8, RNH201, ROG3, ROX3, RPL41B, RPS19B, RPS1B, RPS21A, RPS8A, RSA3, RTT109, SCJ1, SDS23, SEC28, SEC61, SGM1, SGN1, SMP3, SNC2, SNF1, SNO2, SNO4, SNZ2, SPI1, SPT6, SPT8, SSF1, STE24, SYT1, TLG2, UFD1, URA2, UTR1, VAC8, VHS1, VPS24, VPS4, XDJ1, XRN1, YAR044W, YBL083C, YBL086C, YBR259W, YCF1, YCL012W, YCL013W, YCS4, YDL023C, YDL094C, YDR134C, YDR179W-A, YDR537C, YEL057C, YEL059W, YER156C, YGR176W, YJL064W, YJR115W, YKR078W, YLL017W, YLR217W, YMD8, YML090W, YMR316C-A, YNL105W, YNL115C, YNL120C, YNR005C, YOR082C, YOX1, YPR011C, YPR038W, YPR123C, YPT52*
**Negative interaction**[a]	*AAC3, AGA1, AKL1, ALG6, ARC18, ARF1, ASF1, ATG2, ATP10, BIM1, BRE2, BUB1, CAB5, CAF20, CAT5, CBP4, CCC1, CDC1, CDC10, CDC48, CGR1, CIN8, CKI1, CNB1, COX10, COX12, COX7, CSE2, CSG2, CTF4, CTM1, DFG16, DFG5, DMC1, DNM1, DOT1, DOT6, DPH5, DRS2, EMI5, ENT5, ERG6, ESC2, FMT1, FPR1, GID7, GIM4, GLO2, GRS1, GSM1, HAP2, HAP5, HCR1, HMS1, HPA3, HXK1, ICE2, IPL1, JHD2, KAP120, LAS17, LIP2, LPD1, LPX1, LSM1, MAF1, MAK10, MBA1, MBR1, MDH3, MMM1, MNE1, MNN10, MSB4, MSC6, MSN4, MTC6, MUB1, MUM2, NIP100, NOT3, NQM1, NSE3, NUP133, NUP188, OCA1, OST4, PAC10, PBA1, PBP4, PCF11, PEX12, PEX15, PMS1, PMT1, PPM2, PSO2, PUF4, QCR2, QCR9, RAD4, RGD1, RMD1, RMR1, RPL11B, RPL13A, RPL20B, RPL37A, RPL6B, RPS0A, RPS11B, RPS25A, RRM3, RTA1, RTC2, RTR1, RTS3, RVS167, SAC7, SAM1, SCT1, SDL1, SEC28, SEC39, SFI1, SIA1, SIC1, SIF2, SIT1, SLC1, SLP1, SNF1, SOK1, SOL4, SPO21, SPO71, SPT8, SWD1, TAD3, TEX1, TGS1, TPK2, TRI1, VAM6, VID30, VPS24, VPS4, VPS72, VPS9, XPT1, YBR099C, YCL075W, YDR445C, YEL010W, YER087C-A, YER181C, YGL260W, YGL262W, YGR021W, YHC1, YKR033C, YLR252W, YLR290C, YLR407W, YLR428C, YML090W, YNL095C, YNL109W, YNL296W, YNL320W, YOL035C, YOR019W, YOR041C, YPL102C, YPL113C, YPQ2, YPR123C, YPT11, YPT53, YSC83*

[a] normalized growth of double *top2-ts/collection* mutants are compared to that of single collection mutants, both grown at 25ºC.

Table S4. Genes that interact with both *top2-4* and *top2-5* at 25ºC.

**Positive interaction**[a]	*AAD3, ABF1, ABP140, ADE6, ADH2, AFG1, ALG14, ALG8, APC5, APL4, ARP3, CBT1, CDC10, CDC20, CDC21, CDC4, COS111, COX23, CUP2, CWH41, DDI3, DOG2, ENO1, FKS3, FUM1, GAB1, GAD1, GAS1, GAS4, GLC8, GPI2, GSY1, HAP2, HUA1, HYP2, IRC24, ITC1, KTR5, LST4, MFA1, MMM1, MOB2, MSS18, NCS2, OST3, PAH1, PET10, PFY1, POB3, POL5, PRE4, QCR2, RAD14, RAD23, RAD3, RPL21B, RTC5, SCS7, SGV1, SNU13, SPF1, SPT15, STU1, SWM1, TAF12, TED1, TGL3, TMA19, UBP6, XPT1, YBL071C, YBL096C, YDJ1, YDL162C, YDR157W, YEL023C, YEL025C, YGL046W, YGL138C, YGL165C, YGR011W, YHC1, YIL151C, YKL069W, YKL151C, YLR346C, YME1, YMR306C-A, YMR310C, YNL024C, YPR098C, YPR197C*
**Negative interaction**[a]	*ALG8, APL6, APP1, ATP23, BCK1, CDC8, CMC1, COA4, COQ10, COX5A, CSM1, CTP1, DAL81, DBF2, DDI1, DED1, EPS1, FLX1, FSH2, GSH1, HAP4, HAT2, HOM2, HOM6, HTZ1, HUR1, INP52, IZH1, MAS2, MDM35, MOT1, MPS1, MRP49, MRPL1, MSS18, NPR2, NST1, OMS1, PAT1, PMR1, PRP9, QRI1, RMD5, RPN11, RPO41, RPP1A, RPS27B, RPS7B, RTC6, RTS1, RTT103, RVS161, SAC3, SCS2, SEC21, SEC22, SIN3, SMI1, SNF4, SNN1, TAF5, THR1, TOP1, TPS2, TRM7, UME6, VIP1, YDR290W, YHL005C, YJR120W, YLR031W, YLR402W, YNL089C, YOR152C, YPR039W*

[a] normalized growth of double *top2-ts/collection* mutants are compared to that of single collection mutants, both grown at 25ºC.

Table S5. Genes/alleles that interact with *top2-4* in any temperature regime.

Clusters[a]			
25ºC[b]	30ºC[c]	6h x 37ºC[d]	
+	0	0	*ADH6, FUM1, YGL138C, YEL023C, AFG1, YEL025C, MAL31, MLS1, TED1, RAD23, MDH2, ITC1, RPL21B, YBL096C, YKL151C, YGR011W, MTC7, RPL37A, RTC5, GSY1, SCS7, UBA4, PAH1, SPF1, CBT1, CSE2, RPL24B, YME1, YNL024C, HAP2, RAD14, APL4, HHF1, YBL071C, YGL046W, YIL151C, PTH1, TMA19, GAD1, IRC24, XPT1, GAS4, COS111, pre2-v214a, mob2-26, mob2-14, hyp2-1159, mps1-417, gab1-2, rad3-ts14, pre4-ph, mob2-22, pre2-75, scl1-ph, mps1-3796, cdc4-1, OST3, pob3-7, hyp2-1, gpi2-774, lcb1-5, erg11-td, tim44-8, snu13-l67w, YKL069W, cdc4-3, snu13-ph, PET8, ADE6, KTR5, MFA1, HUA1, NCS2, YPR098C, alg14-ph, pol5-2, yhc1-8, cdc123-4*
+	+	+	*YMR306C-A, FKS3, TGL3, ADH2, ELP6, GLC8, UBP15, taf12-w486stop, stu1-5, cdc10-4, abf1-101, SCW10*
0	+	+	*mob2-20, pfy1-14, apc5-ca, esa1-d414, sgv1-35, yhc1-1, cdc21-ts, arp3-f306g, AVT5, DEP1, RAX2, FLD1*
+	-	-	*UTH1, COX23, MSS18, ABP140, CWH41, AAD3, PET122, YPR197C, met30-6, RPS29B, YDJ1, SPA2, RHO4, ALG8, YOL106W, mas2-10*
0	+	0	*npa3-ph, YMR310C, YJL150W, BLI1, YLR063W, PUT2, REX2, YDR133C, SIP4, LST4, YBL059W, DUS3, MSC7, YGR266W, NIT3, ELO1, IPT1, SLX4, ACA1, PIN4, MBA1, PET10, SKI2, YNL134C, SGF73, ECM10, SIP3, YGL152C, YHR022C, PBI2, SLI1, MNS1, DOG2, MIC23, FRE1, RNH203, HKR1, ENO1, GRE2, MDH1, EMP46, INM2, ERG3, YDR157W, NAT5, PCL9, YKL053W, PPH3, TRK2, YKR051W, UFD2, UBP6*
0	0	+	*CTH1, GIR2, SIF2, YGL165C, CUP2, MSB2, IST1, YGR012W, YDR018C, FMP41, YGL015C, LEA1, RSF1, YRB30, EFT1, DDI3, YHR138C, GCV1, ECM23, SDH1, BSP1, DON1, YOR325W, SCW11, RPS6B, YDL162C, YGL159W, SWD3, YPL062W, YOR059C, YOL019W, YFL052W, AFR1, TVP23, SWT1, YMR010W, SWM1, CCC2, nse3-ts4, dbf4-3, cdc20-2, cdc26-1, cdc19-1, pcf11-ts10, cdc37-1, stu2-12, RBD2, YDR391C, SAS4, YKL077W*
-	0	0	*INP52, APT2, TOM7, SNN1, YDR290W, EXG1, RPS7B, TOP1, lcb1-2, DBF2, brl1-3231*
0	-	-	*PET20, SHH4, VPS51, BOP2, YLR108C, YOL029C, YLR241W, STP3, YLR031W, CPR7, SMI1, MCK1, CBR1, APS2, SKG3, NPR2, URC2, VIP1, MHT1, RPA34, RUD3, GRX6, RPL40B, RPL34B, YHR140W, SCS2, RTC6, SDC1, MKS1, UPS2, PAT1, pob3-q308k, cdc21-1, mot1-1033, prp9-ts, SEC22, ALP1, TRM7, ALG8, YOL106W, mas2-10*
-	-	-	*MSH3, RTT103, RTC1, act1-2, CSM1, cdc2-7, YPT7, nop2-9*

0	-	0	*MDM38, YLR402W, HAT2, IME2, SAC3, YML035C-A, YPL034W, RFX1, OSW2, FMP46, RPP1A, SVL3, PHD1, YLR444C, SWC7, EAR1, SAM2, YPR084W, RPS27B, LSM6, YLR391W, MNL2, RNH1, SYC1, AIM26, YCK3, SWA2, YLR269C, PIN2, YNL171C, YDR161W, PUN1, RTC4, ERG5, RPS4B, RRT12, YLR152C, RPS25B, YEL059W, UBC12, TRM1, YCT1, THI7, EPS1, ORM2, ERC1, AIM24, RPL14A, FSH2, TFS1, YHL005C, SLA1, YGR272C, MRPL10, SNA2, SLM4, YLR236C, MGS1, RPO41, NHP10, HIM1, RMD5, YAR1, YPR039W, DEG1, KAR4, RPA14, ECM19, SSO2, YOR062C, MDM35, taf5-20, qri1-ph, RPS29B, YDJ1, SPA2, RHO4*
0	0	-	*ypr086w-ph, DDI1, ATO2, BRE1, AIM18, YOR152C, YGR250C, CTP1, TOS6, YCF1, COA1, PHB2, OPI1, PDC1, PTM1, YNL195C, FMP25, YKR104W, AMD1, ydr416w-ph, cdc8-1, nse4-ts3, rpn11-8, nse5-ts1, sec26-f856aw860a, sec23-1, cic1-2, sec21-1, HAP4, ATP18, MAM3, YNR040W, GSH1, PMD1, VAM7, MKC7, PCL1, PML1, RPS11A, UGA2, UME6, OMS1, FLX1, MRPL1, STP4, YJR120W, THR1, COA4, BCK1, SNF4, THI73, DAL81, HOM2, HMI1, MRP49, HAP3, CMC1, CKB1, HDA1, COQ10, MIG1, TPS2, UBP8, BST1, HOM6, YPL150W, PAN2, RVS161, PEX13, RTS1, YJL021C, GDE1, VRP1, YBR287W, PRM2, GAL83, YDL119C, SHO1, SCY1, MTC3, CWH43, POL32, PMR1, HUR1, COX5A, ATP23, UBP6, UTH1, COX23, MSS18, ABP140, CWH41, AAD3, PET122, YPR197C, met30-6*

[a] + : positive interaction, - : negative interaction, 0: no interaction

[b] normalized growth of double *top2-ts/collection* mutants are compared to that of single mutants in the collections, both grown at 25ºC.

[c] normalized growth of double *top2-ts/collection* mutants grown at 30ºC are compared to the same double mutants grown at 25ºC. For instance, "+" at 25ºC and "+" at 30ºC indicates that there was a positive genetic interaction at 25ºC that was enhanced at 30ºC; "+" at 25ºC and "0" at 30ºC indicates that there was a positive genetic interaction at 25ºC that was maintained to a same degree at 30ºC; "+" at 25ºC and "-" at 30ºC indicates that there was a positive genetic interaction at 25ºC that diminished at 30ºC (either to neutral or negative interaction); "0" at 25ºC and "+" at 30ºC indicates that a positive genetic interaction at 30ºC was seen that did not happen at 25ºC; "0" at 25ºC and "-" at 30ºC indicates that a negative genetic interaction at 30ºC was seen that did not happen at 25ºC; etc.

[d] normalized growth of double *top2-ts/collection* mutants grown first during 6 h at 37ºC and then at 25ºC for 2 d are compared to the same double mutants grown always at 25ºC. The interpretation of the genetic interactions is similar to the above paragraph.

Table S6. Genes/alleles that interact with *top2-5* in any temperature regime.

Clusters[a]			
**25ºC[b]**	**30ºC[c]**	**6h x 37ºC[d]**	
+	0	0	*COS111, GAS4, FUM1, TED1, YEL057C, YDR537C, YMR310C, GAS1, AFG1, YEL025C, RPL21B, YEL023C, YGL138C, EDC3, ITC1, IME2, RTC5, RAD23, FLO1, GSY1, HRQ1, UTR1, PHR1, YNL024C, YIL151C, YGR011W, IRC24, YBL071C, RAD14, GAD1, APL4, ABP140, AAD3, URA2, dbf2-3, ufd1-2, sec61-2, cdc24-1, smp3-1, spt6-14, prp9-1, cop1-1, pre4-ph, pse1-41, sgv1-80, alg14-ph, gab1-2, qri1-ts6, rgr1-100, OST3, snu13-ph, hyp2-1159, RIM8, nop4-3, gpi2-774, cct4-1, pol5-2, brn1-9, cog3-1, abf1-101, snu13-l67w, YKL069W, rox3-182, dcp2-7, cdc53-1, spt15-i143n, rad3-ts14, YPR098C, POC4, APM1, MRS2, YPR197C, COX5B, PSD2, FAA4, ELP4, NCS2, EGD1, HUA1, ADE6, STE24, MFA1, KTR5, AXL1, CBT1, SCS7, FMS1, VAC8, ELP3, MOG1, PAH1, YBL096C, YEL059W, RPS19B, SPF1, YNL120C, MGR2, hyp2-2, TLG2, XRN1, YGL046W, YME1, RPS8A, DOM34*
+	+	+	*DAN1, cdc14-3, mob2-40, pol1-ts, nop1-3, cdc15-1, apc5-ca, cdc21-ts, cdc4-3, hrp1-4, pob3-7, RPL41B, YLR217W, AIM5, YCL013W, YDR179W-A*
0	+	+	*yhc1-1, mob2-26, cdc10-4, mob2-22, taf12-w486stop, stu1-5, YMR306C-A, ADH2, FKS3, YKL151C, TMA19*
+	-	-	*SPT8, SNF1, COX10, COX12, CAT5, QCR2, YML090W, VPS24, MMM1, YPR123C, HAP5, MSS18, HAP2, SEC28, MAF1, XPT1, CGR1, HCR1, VPS4, ALG8, QCR9*
0	+	0	*CGI121, MPH1, SGN1, YNL105W, YOR082C, UBP6, PAC11, YAR044W, MCH1, CSF1, YCF1, YOX1, RPS21A, YMR316C-A, DDI3, SGM1, SSF1, PET10, HDA1, OAF3, ATX1, ABZ2, DOG2, SYT1, PHO91, YDL094C, PHO90, PCK1, YJL064W, YBR259W, PLP1, RAD16, MCK1, ROG3, YDL162C, YDR157W, HNT2, HCH1, ENO1, GAL83, YKR078W, SNZ2, KNS1, LST4, MCX1, YER156C, PUT3, orc2-1, act1-124, myo2-16, pfy1-14, arp3-f306g, PAM17, HOM3, EAR1, RPS1B, TGL3, SNO4, ELM1*
0	0	+	*ACE2, OAR1, MDJ2, ALT1, YNR005C, SCJ1, CUP2, SWM1, YJR115W, DLD1, YCL012W, YGR176W, YPR011C, SDS23, CTS1, CCW12, YNL115C, AVT4, APJ1, YGL165C, SPI1, VHS1, CPR5, YPT52, LAT1, YPR038W, MRP8, IRC8, SNO2, PRC1, YMD8, AIM7, OCA4, YLL017W, OCA6, YDR134C, PUF3, ALD4, IRC6, CLB1, YDL023C, LSC2, CLA4, YBL086C, YBL083C, MEP1, RCY1, lcb2-16, cdc20-2, ELM1, RTT109, RNH201, ATG2*
-	0	0	*RTT103, DBF2, YNL089C, YDR290W, CSM1, YDR445C, ESC2, DOT1, YNL095C, RPS7B*

0	-	-	*LIP2, RPS11B, MDH3, YPT11, HMS1, RMD5, NPR2, DFG16, cdc48-1, IZH1, ATP23, OMS1, SNF4, TEX1, EPS1, RRM3, HTZ1, ERG6, YCL075W, OST4, KAP120, YJR120W, UME6, SIA1, LPD1, HCR1, VPS4, ALG8, QCR9*
0	-	0	*PAC10, TPK2, PBP4, SIT1, HPA3, EMI5, VPS72, SAC3, YLR407W, YNL109W, YOL035C, DFG5, CTF4, PPM2, CCC1, DOT6, RPS27B, YER087C-A, YOR041C, JHD2, YGR021W, DPH5, APL6, RTS3, YKR033C, RAD4, DMC1, TRI1, YLR252W, SLC1, CNB1, SAM1, BIM1, MBR1, CTP1, YBR099C, YLR031W, SDL1, RTA1, RTC2, DRS2, YPR039W, ENT5, PUF4, YOR019W, CIN8, RPS0A, RPL11B, RPO41, RGD1, CAF20, SPO71, MSB4, GID7, VAM6, YHL005C, YER181C, PMS1, SCS2, LSM1, YPL102C, ASF1, SIF2, RPL13A, RTR1, GLO2, RPL37A, FSH2, NUP133, YLR402W, GIM4, RPP1A, HAT2, YEL010W, RPL6B, mas2-10, tad3-ph, prp9-ts, qri1-ph, sec39-ph, taf5-20, cab5-ph, nse3-ts3, pcf11-1, cdc1-4, mot1-1033, ATG2, XPT1, CGR1*
0	0	-	*SCT1, YGL262W, YPL113C, RVS161, COA4, YPQ2, MNN10, YOR152C, SMI1, PSO2, CSE2, SIC1, HUR1, PMR1, COX7, COQ10, HAP4, YNL296W, THR1, TPS2, BCK1, MBA1, MSC6, HOM6, AGA1, MRPL1, MSN4, COX5A, MNE1, TGS1, SWD1, CMC1, MAK10, SOK1, AAC3, BRE2, MUM2, MUB1, SAC7, MRP49, YGL260W, MTC6, SPO21, RTS1, RMR1, FLX1, VID30, CSG2, SLP1, RVS167, GSH1, YLR428C, PEX12, CBP4, FMT1, RPS25A, FPR1, NIP100, DNM1, las17-1, rpn11-8, sec21-1, ipl1-1, cdc8-1, PBA1, RPL20B, HOM2, NUP188, SEC22, ARF1, RTC6, YNL320W, ICE2, SOL4, HXK1, BUB1, YLR290C, YSC83, VIP1, CKI1, CTM1, LPX1, NQM1, PEX15, NOT3, MDM35, VPS9, TRM7, AKL1, DDI1, GRS1, GSM1, DAL81, RMD1, ATP10*

[a] + : positive interaction, - : negative interaction, 0: no interaction

[b] normalized growth of double *top2-ts/collection* mutants are compared to that of single mutants in the collections, both grown at 25ºC.

[c] normalized growth of double *top2-ts/collection* mutants grown at 30ºC are compared to the same double mutants grown at 25ºC. For instance, "+" at 25ºC and "+" at 30ºC indicates that there was a positive genetic interaction at 25ºC that was enhanced at 30ºC; "+" at 25ºC and "0" at 30ºC indicates that there was a positive genetic interaction at 25ºC that was maintained to a same degree at 30ºC; "+" at 25ºC and "-" at 30ºC indicates that there was a positive genetic interaction at 25ºC that diminished at 30ºC (either to neutral or negative interaction); "0" at 25ºC and "+" at 30ºC indicates that a positive genetic interaction at 30ºC was seen that did not happen at 25ºC; "0" at 25ºC and "-" at 30ºC indicates that a negative genetic interaction at 30ºC was seen that did not happen at 25ºC; etc.

[d] normalized growth of double *top2-ts/collection* mutants grown first during 6 h at 37ºC and then at 25ºC for 2 d are compared to the same double mutants grown always at 25ºC. The interpretation of the genetic interactions is similar to the above paragraph.

LEGENDS TO EXCEL FILES INCLUDED IN FILE S2.zip

File: **SGAtool data_top2-4_TSv6_RT.xlsx**
Raw and processed SGA data of the permissive (25º) experiment where *top2-4* was the query allele used against the collection of thermosensitive alleles.

File: **SGAtool data_top2-4_TSv6_30C.xlsx**
Raw and processed SGA data of Top2 constant downregulation (30º) experiment where *top2-4* was the query allele used against the collection of thermosensitive alleles.

File: **SGAtool data_top2-4_TSv6_37Cx6h.xlsx**
Raw and processed SGA data of the transient Top2 inactivation (37º x 6h) experiment where *top2-4* was the query allele used against the collection of thermosensitive alleles.

File: **SGAtool data_top2-4_DMA_RT.xlsx**
Raw and processed SGA data of the permissive (25º) experiment where top2-4 was the query allele used against the collection of gene deletions.

File: **SGAtool data_top2-4_DMA_30C.xlsx**
Raw and processed SGA data of Top2 constant downregulation (30º) experiment where *top2-4* was the query allele used against the collection of gene deletions.

File: **SGAtool data_top2-4_DMA_37Cx6h.xlsx**
Raw and processed SGA data of the transient Top2 inactivation (37º x 6h) experiment where *top2-4* was the query allele used against the collection of gene deletions.

File: **SGAtool data_top2-5_TSv6_RT.xlsx**
Raw and processed SGA data of the permissive (25º) experiment where *top2-5* was the query allele used against the collection of thermosensitive alleles.

File: **SGAtool data_top2-5_TSv6_30C.xlsx**
Raw and processed SGA data of Top2 constant downregulation (30º) experiment where *top2-5* was the query allele used against the collection of thermosensitive alleles.

File: **SGAtool data_top2-5_TSv6_37Cx6h.xlsx**
Raw and processed SGA data of the transient Top2 inactivation (37º x 6h) experiment where *top2-5* was the query allele used against the collection of thermosensitive alleles.

File: **SGAtool data_top2-5_DMA_RT.xlsx**
Raw and processed SGA data of the permissive (25º) experiment where *top2-5* was the query allele used against the collection of gene deletions.

File: **SGAtool data_top2-5_DMA_30C.xlsx**

Raw and processed SGA data of the Top2 constant downregulation (30º) experiment where *top2-5* was the query allele used against the collection of gene deletions.

File: **SGAtool data_top2-5_DMA_37Cx6h.xlsx**

Raw and processed SGA data of the transient Top2 inactivation (37º x 6h) experiment where *top2-5* was the query allele used against the collection of gene deletions.
